# Ontogenetic Sequence of Differential Gene Expression in Predator‐Induced *Daphnia pulex*

**DOI:** 10.1111/mec.70446

**Published:** 2026-07-01

**Authors:** Andrey Rozenberg, Linda C. Weiss, Tatjana Schwarz, Nancy Kühne, Uwe John, Ralph Tollrian

**Affiliations:** ^1^ Department of Animal Ecology, Evolution and Biodiversity Ruhr University Bochum Bochum Germany; ^2^ The Technion—Israel Institute of Technology Haifa Israel; ^3^ Alfred Wegener Institute Bremerhaven Germany

## Abstract

In response to predators, many *Daphnia* species develop inducible morphological defenses. These traits are phenotypically plastic, meaning their production is suppressed in the absence of the predator. While previous studies have identified several neurohumoral factors and candidate genes involved in the development of the defenses, the involvement of specific genes and factors remains uncertain, and minimal information is available about the timing of gene expression changes during development. In the current study, we thus performed a candidate‐independent gene expression analysis of defense development over juvenile developmental stages in *D. pulex* . We analyzed transcriptome responses of the microcrustaceans to the natural mix of predator‐emitted compounds and also to the recently identified pure kairomone synthesized in the lab. With the data obtained, we show that the main factor correlating with the global gene expression patterns in *Daphnia* is molt cycle. Influence of kairomone treatment on the global gene expression is evident only in certain stages. The different stages generally show unique patterns of kairomone‐induced gene expression. However, expression profiles are similar for the naturally released and the chemically synthesized kairomone in each one of the stages. A number of genes with regulatory, structural and detoxification roles are differentially expressed as a reaction to the kairomone treatment. The most consistent response was found in the expression levels of *ilp‐3* coding for an insulin‐like peptide. Gene knockdown experiments suggest that this hormone plays a role in the production of the defense. Many of the genes responding to the kairomone treatment have no predicted function, stressing the need to investigate gene functions in *Daphnia*.

## 1 Introduction

*Daphnia* microcrustaceans adapt their life cycle, behavior and/or morphology when exposed to predation (Tollrian and Dodson 1999). Most of these changes protect against predator attacks, but their production comes at a cost that is avoided in the absence of the predator (Riessen 1992; Tollrian and Harvell 1999). *Daphnia*'s defensive mechanisms are very diverse and often involve easily detectable morphological alterations in the form of elongated spines, helmets, crests, as well as small cuticular neckteeth or the peculiar ‘crown of thorns’ (Tollrian and Dodson 1999; Petrusek et al. 2009). Some of these morphological structures emerged several times in the evolution of the genus. Thus, the neckteeth developing under predation by the phantom midge larvae *Chaoborus* are characteristic to several not directly related lineages of *Daphnia* including the *D. pulex* group (Colbourne et al. 1997; Tollrian and Dodson 1999; Kotov et al. 2006; Juračka et al. 2011).

Information about the predator's presence is mediated mainly by chemical cues unintentionally produced and released by the predator, referred to as kairomones (Tollrian and Dodson 1999). Although attempts at isolation and characterization of kairomones with physiological effects on *Daphnia* have been undertaken since the 1990s (Parejko and Dodson 1990; Tollrian and von Elert 1994; Von Elert and Loose 1996; Akkas et al. 2010), the identity of most kairomones capable of inducing physiological reactions in *Daphnia* remains unknown. Among the few kairomones whose precise chemical structures have been elucidated, one of the best‐characterized is the fish kairomone 5α‐cyprinol sulfate: this bile salt was identified as the active compound inducing diel vertical migration in *Daphnia* (Hahn et al. 2019). Similarly, the kairomones emitted by *Chaoborus* have been chemically identified and experimentally validated (Weiss et al. 2018). The *Chaoborus* kairomone consists of a family of long‐chained (≥ C14) fatty acids conjugated to the α‐amine of L‐glutamine. We previously identified six structurally similar compounds within this mixture that are biologically active and induce neckteeth formation in *D. pulex* (Weiss et al. 2018). Among them, N‐oleoyl L‐glutamine was the most abundant, although all identified components showed comparable potency.

Notwithstanding the diversity of the defensive structures in diverse *Daphnia*, they share the same underlying cellular mechanism associated with a specific population of large polyploid cells in the corresponding head regions (Beaton and Hebert 1997; Weiss, Tollrian, et al. 2012; Weiss et al. 2015). Moreover, the same set of neurohumorally active compounds: dopamine and acetylcholine, is involved in the formation of the structurally dissimilar neckteeth and crests in *D. pulex* and *D. longicephala*, respectively (Weiss et al. 2015). At least in *D. pulex* , juvenile hormone (Miyakawa et al. 2010, 2013) is seemingly also involved in the regulation of the neckteeth production, although a subsequent study failed to reproduce results of some of the earlier qPCR experiments (Christjani et al. 2016). Based on gene expression data, the life history shifts accompanying the neckteeth production were connected to ecdysone (Miyakawa et al. 2010; Dennis et al. 2014), although the role of ecdysone has also been questioned by Christjani et al. (2016). Neckteeth development and the overall thickening of the cuticle were shown to be correlated with up‐regulation of several cuticle‐associated proteins (Rozenberg et al. 2015; Christjani et al. 2016; An et al. 2018), even though temporal and spatial patterns of their expression remain to be explored. Little is known about the early steps of kairomone perception: glutamate receptors were shown to be up‐regulated in the embryos shortly after kairomone exposure (Miyakawa et al. 2015; An et al. 2018), and their antagonists were shown to inhibit formation of the neckteeth (Miyakawa et al. 2015), but the kairomone receptor itself remains unidentified.

**FIGURE 1:**
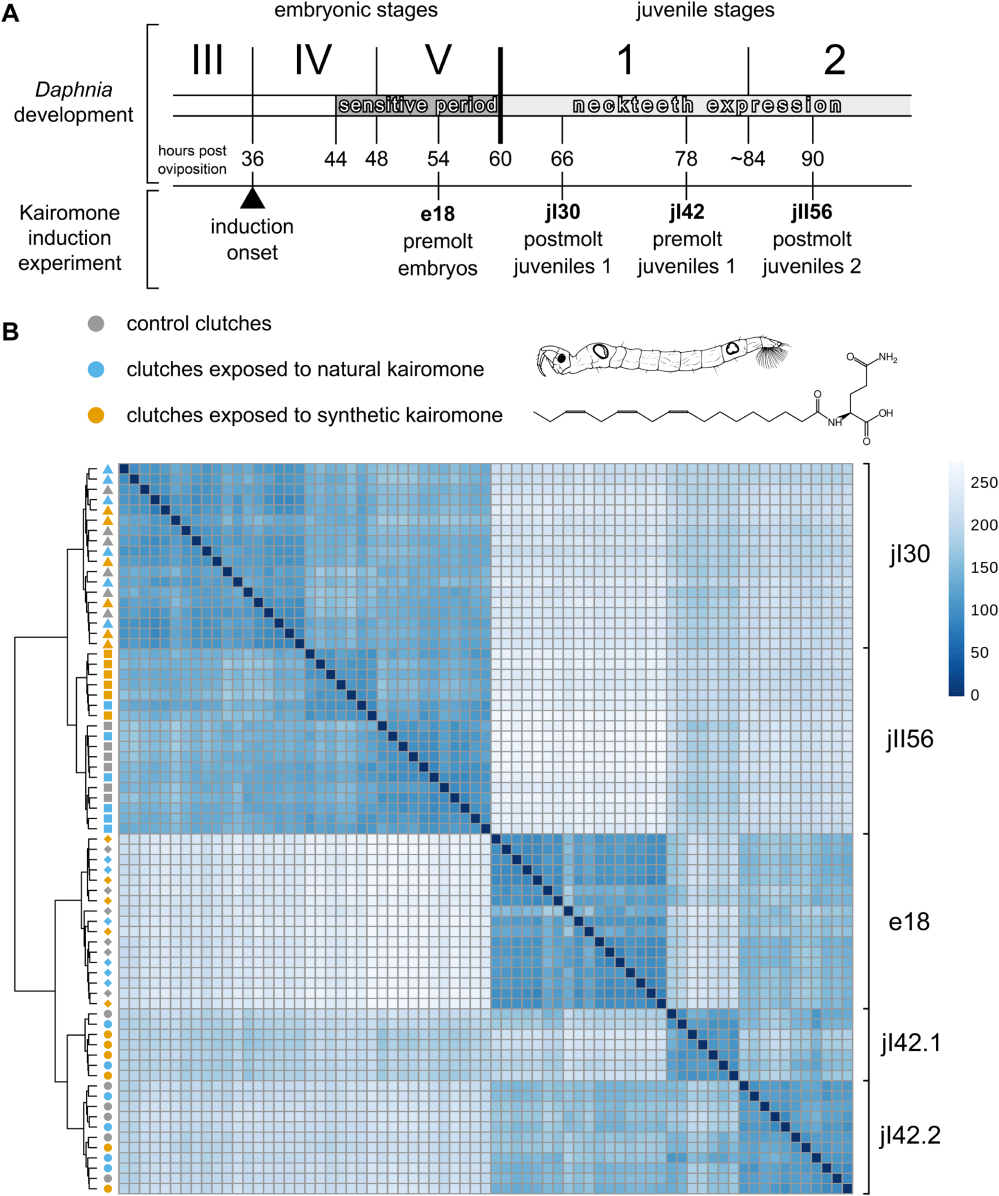
An overview of the kairomone‐induction experiment used to generate Tag‐Seq libraries. (A) Experimental setup utilized in the current study in the context of *Daphnia pulex* development. Morphological stages are classified according to Tollrian (1994) and are based on easily‐recognizable landmarks, but do not directly correspond to intervals between embryonic molts (Weiss et al. 2016; Kotov and Boikova 2001) shown above as dashed arrows. Solid arrows denote post‐embryonic exuviations, the first of which occurs within 20–30 min after neonates leave their mother's brood pouch (Kotov 1997; Mittmann et al. 2014). The kairomone timing bar shows the kairomone‐sensitive period (Naraki et al. 2013; Weiss et al. 2016) (dark gray) and the period of neckteeth expression (light gray). Experiments performed in this study were initiated with the appearance of eyes in embryos, 8 h before the onset of the sensitive period. Four time points were sampled after 18 h with an interval of 12 h. A single sample corresponded to a single clutch of siblings. (B) Heatmap and hierarchical clustering of the *Daphnia* Tag‐Seq libraries according to overall expression patterns (rlog‐normalized data). Sample names denote stage (‘e’ for embryo, ‘jI’ for juvenile 1 and ‘jII’ for juvenile 2), time since induction onset in hours, and experimental condition. The outlier sample e18fs1 is omitted from the plot.

Given the conflicting evidence of the role of some of the neurohumoral factors in the production of the defenses even in the best studied system *D. pulex* –*Chaoborus* (Christjani et al. 2016), as well as the fact that every next study finds new regulators and new target genes (Miyakawa et al. 2015; Rozenberg et al. 2015), one might conclude that the understanding of defense development is far from complete. These considerations motivated us to perform a replicated time‐series study of gene expression using a candidate‐independent approach. We chose to focus on four distinct time points covering the developmental interval from kairomone perception to the presumptive decline of its effect (Figure 1A). As other compounds co‐produced by the predator alongside the kairomone, as well as alarm cues from the prey (Laforsch et al. 2006), might be physiologically active and consequently influence genetic responses, we decided to perform two sets of treatment experiments: the kairomone as part of the natural mix of predator‐associated compounds and the chemically synthesized pure kairomone. To increase statistical power of the differential gene expression analysis, each combination of treatment/stage was performed in six biological replicates, with libraries prepared for individual clutches. The focus on single clutches allowed us to achieve a precise synchronization between individuals in the same sample, which, as we demonstrate, is crucial to accounting for life‐history changes. Gene expression assessment was carried out following our previously published Tag‐Seq protocol (Rozenberg et al. 2016).

## 2 Materials and Methods

### 2.1 Material and Experimental Design

The clone R9 used for the experiments belongs to the species referred to as North American or Pan‐Arctic *Daphnia* ‘*pulex*’ and was originally isolated from Ontario province, Canada. The general scheme of the experiment and its relation to *D. pulex* development are shown in Figure 1A. Animals were propagated strictly parthenogenetically in 10 L buckets with aged charcoal‐filtered tap water under constant light and fed with *Chlorella vulgaris* microalgae *ad libitum* (< 1.5 gC/L). For seven generations before the onset of the experiment, every next generation was initiated from juveniles of the third or later clutch to decrease inter‐individual variation. After the females of the eighth generation released their third‐fourth clutch, their reproductive cycle was monitored on an individual basis. 72 females in total were selected when embryos in their brood pouches reached the early fourth stage (Tollrian 1994), which is a morphologically easily recognizable time point 8 h before the onset of the kairomone‐sensitive period (Weiss et al. 2016) (Figure 1A). Each female was placed individually in a 50 mL glass vial with one of the three following media:

1. Control: same water as used for the culture, void of predator cues.
2. Natural kairomone: 1:5 dilution of phantom midge larvae‐exposed water in the water used for the culture. The stock solution was prepared by placing 50 *Chaoborus* larvae fed on 500 juvenile R9 *Daphnia* in a 1 L jar for 24 h. The stock was prepared in two batches, filtered using a GF/C‐grade filter, mixed and frozen as one‐time aliquots at −20°C until use.
3. Synthetic kairomone (Weiss et al. 2018): 1:25,000 dilution of a stock solution of N‐linolenoyl‐L‐glutamine. The stock was prepared by dissolving the chemically synthesized compound in a 1:10 dioxane:ethanol mixture to a concentration of 12.2 mM.

As the synthetic kairomone medium contained minute amounts of dioxane and ethanol, equal volume of the same solvent mixture was also added to the two other media. Prior experiments showed no adverse effects of such small amounts of ethanol on *Daphnia*'s development or appearance (Weiss et al. 2015). Similarly, no abnormalities were found in the swimming behavior of the juveniles during the course of the experiment and no developmental defects were observed when juveniles were inspected under a stereo microscope before fixation. All media were mixed well before placing the animals. For nourishment, *C. vulgaris* to the final concentration of 1.5 mg carbon/L was added in each vial (stock concentrations were calculated by calibrated photometric measurements).

Twenty‐four hours after the onset of the experiment, females released the neonates (see Figure 1A), which were placed in new vials, one clutch per vial, with fresh media having the same amounts of the components. The juveniles of the first instar molted to the second instar approximately 24 h post‐release.

The experimental animals were sampled at four time points (see Figure 1A): pre‐release embryos 18 h after induction onset (e18), juveniles of the 1st instar after 30 h (jI30), juveniles of the 1st instar after 42 h (jI42) and juveniles of the 2nd instar after 56 h (jII56). Each of the treatment‐stage combinations was replicated six times, with individual clutches assigned as random as possible. The females were discarded after collection of the embryos or transfer of the neonates, and thus every sampled clutch originated from a different female.

Before fixation, the juveniles were controlled for the absence or presence of the neckteeth in the control and induction media, respectively. Each clutch was fixed separately in the RLT buffer from the RNeasy Micro Kit (Qiagen). Mean (±SD) number of progeny per clutch amounted to 21.1 ± 4.5, but no more than 18 individuals were sampled to reduce the variation between the clutches (resulting in 17.2 ± 1.9 individuals per clutch sampled). Upon fixation, the tubes were stored at 4°C overnight and frozen away at −80°C afterwards.

### 2.2 Tag‐Seq Library Preparation

The general protocol utilized to produce the Tag‐Seq libraries is described in detail in Rozenberg et al. (2016). Total RNA was extracted with RNeasy Micro Kit (Qiagen) without the DNase treatment step, ERCC ExFold RNA spike‐in mixes (ThermoFisher) were added (1 μL of 1:100 dilution of the corresponding mix per 500 ng total RNA) and the RNA was fragmented by hydrolysis. The polyA‐tail bearing RNA species were captured with oligo‐dT beads and ligated to P5 adapters containing moderately degenerate base regions (mDBR) to later allow demultiplexing and deduplication of the data. First‐strand cDNA was obtained from an oligo‐dT‐VN primer and used as template in a 10‐cycle PCR with one of the primers tailed with the second, regular index (see Data S1 for the list of the P5 and P7 indexes). Libraries to be run on the same flowcell were pooled, co‐purified and size‐selected in the range: 270–500 bp on LabChip XTe with DNA 750 Assay Kit (PerkinElmer). The broad size range of the selected fragments was achieved by combining two narrower size intervals which resulted in a two‐peaked distribution of fragment sizes. The pooling was performed equimolarly, with the exception that for the embryonic stage the amount of the PCR product taken was increased 1.5 times relative to the other samples to increase sensitivity to the potentially subtler differences in expression levels.

The samples were processed in three batches, and in total three High‐Output 75‐cycles single‐end runs were performed on an Illumina NextSeq 500 machine. Batch and run assignment for individual samples was randomized. See Data S1 for details on sample indexing and read numbers.

### 2.3 Reference Transcriptome

Reference genome assembly of the conspecific clone KAP4 of North American *D. pulex* (RefSeq assembly GCF_021134715.1) was used as the basis for gene quantification. To obtain a more comprehensive gene set, we recruited four unstranded RNA‐Seq sequencing paired‐end Illumina runs previously obtained by us from two bulk *Chaoborus* kairomone induction experiments on strain R9 (SRA numbers SRX1070836, SRX1070837, SRR32573570 and SRR32569538). The RNA‐Seq reads were processed with fastp v. 0.23.2 (Chen et al. 2018) for trimming, deduplication and merging of overlapping read pairs. The resulting read pairs, merged reads and singleton reads were mapped to the KAP4 genome with HISAT2 v. 2.2.1 (Kim et al. 2019) and assembled with StringTie v. 2.2.3 (Pertea et al. 2015) in the references‐guided mode.

For the purpose of phylogenetic analyses, a clone‐specific transcriptome assembly was obtained with SPAdes v. 3.14.1 (Bushmanova et al. 2019) using the fastp‐processed RNA‐Seq data from strain R9.

### 2.4 Data Processing and Analysis

Cutadapt v. 1.9.1 (Martin 2011) was used for quality, adapter and polyA trimming, retaining reads at least 35 nt long. Trimmed reads were demultiplexed and deduplicated with tagseq v. 0.2 (Rozenberg et al. 2016).

Gene quantification was performed with Salmon v. 1.10.2 (Patro et al. 2017) using the transcript sequences from the reference KAP4 transcriptome extended by recruiting the R9 RNA‐Seq data as described above. To define transcript groups distinguishable with the generated Tag‐Seq data, we performed transcript clustering with Terminus v. 0.1 (Sarkar et al. 2020) with a minimum spread value of 0.05, tolerance of 0.01 and consensus threshold of 0.25. Transcript groups were named after the representative transcript with a suffix indicating the type of relationship within the group: ‘~GN’ for groups corresponding to entire genomic loci, ‘~TR’ for single transcripts from loci in which different transcripts belong to different groups, ‘~GNs’ for groups covering all transcripts from more than one genomic locus and ‘~TRs’ for other, more complex cases. Results of the gene expression quantifications are provided in Data S2.

The quantifications were pre‐filtered to include genes with at least 50 read counts in a total of at least six samples and the differential expression analysis was conducted with DESeq2 v. 1.42.0 (Love et al. 2014) with the focus on the effect of kairomone treatments at different developmental stages. The kairomone treatments were coded as two binary variables for the synthetic and the natural kairomone, respectively. To account for the asynchrony in the molting cycle between clutches from the same stage, a molting phase covariate term was added. It was created by taking the first principal component of VST‐normalized expression levels of the chitin synthase 1 gene *kkv* (gene LOC124192393 = XM_046585635.1~GN) and the 7‐dehydrocholesterol desaturase *nvd1* (LOC124192434 = XM_046585704.1~GN) (Figure S1A). To ensure this measure was independent of stage, we residualized the stage contribution using a linear model. Differential gene expression analysis was performed for each developmental stage independently in a two‐stage testing procedure using stageR v. 1.24.0 (Van den Berge et al. 2017) (Figure S1B). In the screening stage, likelihood‐ratio test (LRT) was performed for genes passing filterByExpr from edgeR v. 4.0.16 (Robinson et al. 2009) to test the general effect of the kairomone treatments. The full design formula included the molting phase covariate and the kairomone treatments. During the confirmation phase, we used LRT to test the contribution of the two individual kairomone treatments. Passing the FDR threshold of 0.05 in the confirmation phase and showing an apeglm‐shrunken (Zhu et al. 2019) log_2_‐fold change of at least 0.5 by absolute value was considered as strong evidence of differential expression for a gene in a specific stage under a specific kairomone treatment. Genes for which at least one of the two treatments satisfied the strong evidence criteria and the second treatment passed a more lenient criterion of uncorrected *p*‐value threshold of 0.01, were considered as differentially expressed in both kairomone treatments in a specific stage (see Figure S1B). Results of the differential gene expression analysis can be found in Data S3. Gene expression patterns were analyzed with DEGreport v. 1.38.4 (Pantano 2023).

In order to compare the lists of the differentially expressed genes to the results obtained by An et al. (2018) for *D. pulex* × *pulicaria* TRO using JGI's v1.1 gene models for *D. pulex* TCO, we mapped the corresponding transcripts to the reference transcriptome (see above) using minimap2 v. 2.28 (Li 2018) and considered matches of at least 90% nucleotide identity and at least 50% coverage of the query as orthologs.

### 2.5 Gene Function Analysis

Protein domains and families were predicted for KAP4 proteins with InterProScan v. 5.61‐93.0 (Jones et al. 2014). Over‐representation and gene‐set enrichment analyses were performed with ClusterProfiler v. 4.8.1 (Wu et al. 2021).

### 2.6 Reference Branchiopod Assemblies

For gene phylogenies and LTR prediction, representative genome and transcriptome assemblies from Branchiopoda were recruited. Whenever gene predictions were not available in the source, TransDecoder v. 5.5.0 (Haas 2018) was used to predict coding sequences in transcriptome assemblies (including the R9 transcriptome assembly) and GeneMark‐ES v. 4.62 (Lomsadze et al. 2005) in self‐training mode was used to annotate genes in genome assemblies.

### 2.7 Phylogenetic Analysis of Insulin‐Like Peptides

For phylogenetic reconstruction of *Daphnia*'s ILP‐1/3/4 clade, amino acid sequences of *D. pulex* KAP4 ILP‐1 (RefSeq accession XP_046446612.1), ILP‐3 (XP_046448317.1) and ILP‐4 (XP_046448221.1) were used as BlastP v. 2.15.0 (Altschul et al. 1990) queries against the protein sequences from the reference branchiopod assemblies (*E*‐value threshold of 1E‐03). For each assembly, matching sequences at least 100 residues in length were extracted and clustered at 95% identity level to exclude minor allelic variants with CD‐HIT v. 4.8.1 (Li and Godzik 2006). The collected sequences were combined, aligned with MAFFT v. 7.520 (Katoh and Standley 2013) in automatic mode, trimmed with trimAl v. 1.4.1 (Capella‐Gutiérrez et al. 2009) to exclude alignment positions with ≥ 10% gaps and maximum likelihood phylogeny was reconstructed with IQ‐TREE v. 2.4.0 (Minh et al. 2020) with 1000 ultrafast bootstrap replicates (Minh et al. 2013). JTTDCMut+I+R2 was automatically selected as the best‐fit model according to the Bayesian information criterion (BIC). As the monophyly of ILP‐3 from *Daphnia* (*Daphnia*) and *Daphnia* (*Ctenodaphnia*) was not recovered in preliminary analyses despite strong synteny, it was enforced as a constraint.

### 2.8 Analysis of Long‐Terminal Repeat Elements

To collect and annotate long‐terminal repeat (LTR) elements related to the kairomone‐responsive LTR element, the following strategy was deployed. The gag protein sequence of the kairomone‐responsive LTR element from *D. pulex* strain R9 was used as a tBlastN query to locate scaffolds containing related *gag* genes in other Cladocera with an *E*‐value threshold of 1E‐08. gag matches passing the empirical bit‐score threshold of 45 were used to extract the *gag*‐containing regions by adding up to 3000 nt upstream and 12,000 nt downstream to the coordinates of the tBlastN match and excluding too short fragments (< 1600 nt). LTRs were annotated with LTRharvest (Ellinghaus et al. 2008) and both LTR elements and matching fragments lacking LTRs were annotated with LTRdigest (Steinbiss et al. 2009), both from GenomeTools v. 1.2.1 (Gremme et al. 2013). For gene annotation with LTRdigest, protein profiles distributed via GyDB (Lloréns et al. 2008) were used, in addition to two lineage‐specific protein profiles for gag and Asp protease, with a HMMER E‐value threshold of 1E‐05. To create the lineage‐specific profiles, R9 gag and Asp protease protein sequences from the R9 kairomone‐responsive element were used as queries in tBlastN searches against branchiopod assemblies, as described above for gag, and aligned parts of the subject sequences were extracted, aligned with MAFFT, and protein profiles were created with hmmbuild from HMMER v. 3.4 (Eddy 2011).

For phylogenetic analysis, complete *gag* genes (with start and stop codons, without frameshifts and coding for proteins at least 150 amino acids) were extracted from the annotated branchiopod LTR elements. The resulting protein sequences were combined with outgroup sequences: Arc proteins from human, rat, mouse, chicken and fruit fly, and gag protein of the yeast Ty3 retrotransposon. The sequences were aligned with MAFFT and trimmed with trimAl to remove positions with ≥ 10% gaps. In parallel, CD‐HIT was used to cluster the sequences at 90% identity and the gag proteins coming from the most complete genetic elements per assembly were picked from the trimmed alignment as cluster representatives. Maximum likelihood phylogeny was reconstructed with IQ‐TREE with 1000 ultra‐fast bootstrap replicates. JTT+G4 model was automatically selected as the best‐fit model according to BIC.

### 2.9 Gene Knockdown

Gene‐specific double‐stranded RNAi probes for the insulin‐like peptide transcript were generated from *D. pulex* R9 cDNA using specific primers with T7 promoter overhangs (Table S1). Potential off‐targets were searched for using NCBI blast. The highest similarity was obtained for the related *ilp‐4* gene with an overall nucleotide identity of 55% in the region of the ILP_3_1 probe and 81% in the ILP_3_2 region. For ILP_3_1, the longest stretch of residues identical to *ilp‐4* amounted to 16 bp, below the values known to cause off‐target effects (Kulkarni et al. 2006), while for ILP_3_2 it was 20 bp, indicating that it has the potential to elicit knockdown of *ilp‐4*. For this reason, ILP_3_2 was used as an alternative probe to supplement the results obtained with ILP_3_1.

dsRNA synthesis was performed using the T7 RiboMAX Express Large‐Scale RNA Production System (Promega). The resulting probes were administered with Lipofectamine 3000 transfection reagent (Thermofisher). As a control we used a 300 bp eGFP probe (Eupheria, Germany). Two solutions were prepared according to the manufacturer's protocol. Solution A consisted of 25.25 μL phosphate‐buffered saline (PBS; pH 7.4, 0.1 M) and 0.75 μL Lipofectamine 3000 reagent. Solution B contained 16.6 μL dsRNA (1500 ng/μL), 8.4 μL nuclease‐free water, and 1 μL Lipofectamine 3000 reagent. The two solutions were combined to form the transfection mixture.

The transfection mixture was added to designated wells of a 24‐well plate containing 2 mL of Aachener *Daphnia* Medium (ADaM; Klüttgen et al. 1994) or kairomone‐enriched medium, which was prepared by exposing the medium to 10 phantom midge larvae fed with 100 *Daphnia* juveniles over 24 h. We included three conditions: (1) a control without kairomones and without dsRNAi probes, (2) a kairomone treatment with the eGFP control dsRNAi probe and (3) a treatment combining one of the target gene dsRNAi probes with kairomone stimulation. For each group, one *D. pulex* mother with embryos in the red‐eye stage (Weiss et al. 2016) was placed into a well. Once the neonates were released from the brood pouch, they were transferred individually into fresh wells containing the medium and the corresponding dsRNAi mixture. Neckteeth expression was observed in the first and second juvenile instars using a stereo microscope (Olympus SZX 16) equipped with a digital camera controlled by TSO Vidmess software (version 3). Neckteeth were scored according to the criteria described by Tollrian (1993).

Effectiveness of the knockdown was validated via reverse transcription quantitative PCR (RT‐qPCR) of the genes of interest. For that, animals were exposed to the respective probes as described above and collected for qPCR in the first juvenile instar in RNA/DNA shield (Zymo Research). RNA was extracted using the Quick‐RNA Microprep Kit (Zymo) according to the manufacturer's protocol. qPCR reactions were run with a Luna reverse transcription qPCR kit (New England Biolabs, Germany) containing 10 ng/μL RNA. We performed three biological and two technical replicates using *Stx16* as a reference gene as described by Spanier et al. (2010). ∆∆*C*_T_ analysis was performed using the R package PCR v. 1.2.2 (Ahmed and Kim 2018). Significant differences in gene expression were assessed using Student's *t*‐test.

## 3 Results

### 3.1 Study Design and Overview of the Data

A total of 72 Tag‐Seq libraries were prepared with each sample corresponding to an individual *Daphnia* clutch assigned to one of the four developmental stages from late embryo to juvenile 2 and one of the three conditions: control and two kairomone treatments, natural and synthetic (Figure 1A). After raw data quality filtering and demultiplexing, on average 13,130,897 ± 4,620,673 reads (±SD) were retained per sample. With the chosen filtering settings, 88.7% ± 3.6% of the reads per sample remained after PCR duplicate removal, of which 93.7% ± 1.6% could be mapped to the reference transcriptome (Data S1, Figure S2). Analysis of the read counts assigned to spike‐in transcripts demonstrated that the approach was quantitative and reproducible (Figure S3).

### 3.2 Sample Clustering

**FIGURE 2:**
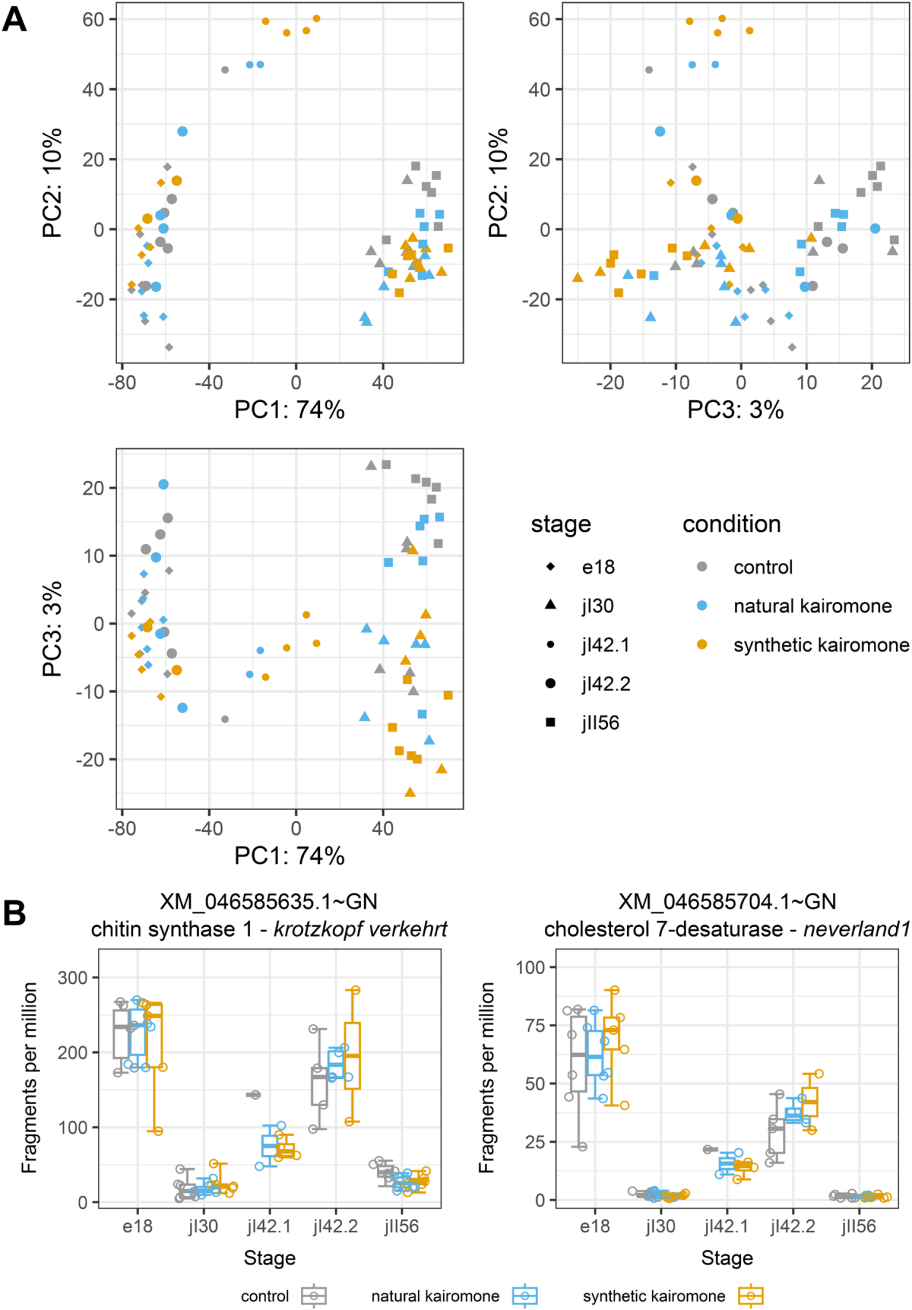
The main source of variation among the *Daphnia* clutches in the kairomone induction experiment is developmental stage and molting phase. (A) Ordination of the Tag‐Seq samples in the axes of principal components based on expression levels of the 500 most variable genes. (B) Expression levels of the markers of molting cycle: Chitin synthase 1 (*krotzkopf verkehrt*) and cholesterol 7‐desaturase (*neverland1*). Whiskers denote min‐max range, boxes—25%–75% quantiles, middle line—median.

Unsupervised clustering of the samples based on *Daphnia* gene expression levels showed that the prevailing factor explaining the between‐sample variability was developmental stage (Figure 1B). Each sample grouped within one of the four clusters corresponding strictly to the respective stage. Despite clustering together with the other e18 samples, sample e18fs1 appeared as an extreme outlier in principle component analysis (PCA) indicating that the corresponding clutch might have contained maldeveloping embryos and was excluded. Clustering analysis and PCA also revealed the jI42 stage to be heterogeneous with two subpopulations designated jI42.1 and jI42.2 (Figures 1B and 2A). Whereas the first principal component in PCA corresponded to the divide between premolt (e18 and jI42.2) and postmolt (jI30 and jII56) stages, the jI42.1 samples occupied an intermediate position in this axis and their separation from the rest of the samples was further explained by the second principal component (see Figure 2A). Expression levels of two markers of the premolting phase: the chitin synthase 1 gene *krotzkopf verkehrt* (Havemann et al. 2008) and the 7‐dehydrocholesterol desaturase *neverland1* (Sumiya et al. 2016), demonstrated a sharp rise in expression levels in the premolt stages e18 and j42.2 contrasting with the low background expression levels in jI30 and jII56, with the juveniles jI42.1 again occupying an intermediate position (Figure 2B). Taken together, this indicates that jI42.1 clutches represent a developmental phase less advanced than jI42.2, hinting at the possibility that at around this time point (78 h post‐oviposition), *Daphnia* undergo a developmental transition leading to two distinct yet temporally very close sub‐stages.

No clear separation of the treatments within the developmental stages was evident in the clustering pattern of the samples, with the exception of the jII56 stage, which was subdivided into two groups: all synthetic‐kairomone‐treated and one natural‐kairomone‐treated clutch in one group and all of the control and the rest of the natural‐hormone‐treated clutches in the other one, a division also explained by the third principal component in PCA (see Figure 2A). We also noticed that the jI42.1 and jI42.2 sub‐stages included unequal proportions of treated and untreated clutches with only a single control jI42.1 clutch (see Figures 1B and 2A). To avoid spurious results, for the downstream analyses we only took the jI42.2 clutches to represent this stage. Overall, we conclude that kairomone induction does not lead to large perturbations in gene expression levels in the earlier stages. In the second juvenile instar, induction with the synthetic kairomone, and to a lesser degree with the natural kairomone, leads to a significant departure in the development of the juveniles from the control. Our results also hint at the possibility that the kairomone induction might delay the jI42.1 > jI42.2 transition, although larger sample sizes are required to test this hypothesis explicitly. To account for the potential remaining effects of the kairomone treatments on the progression of the molting cycle, especially in the later stages, we included a molting cycle covariate in the model used for differential gene expression analysis (see Figure S1A).

### 3.3 General Patterns of Kairomone‐Induced Differential Gene Expression

**FIGURE 3:**
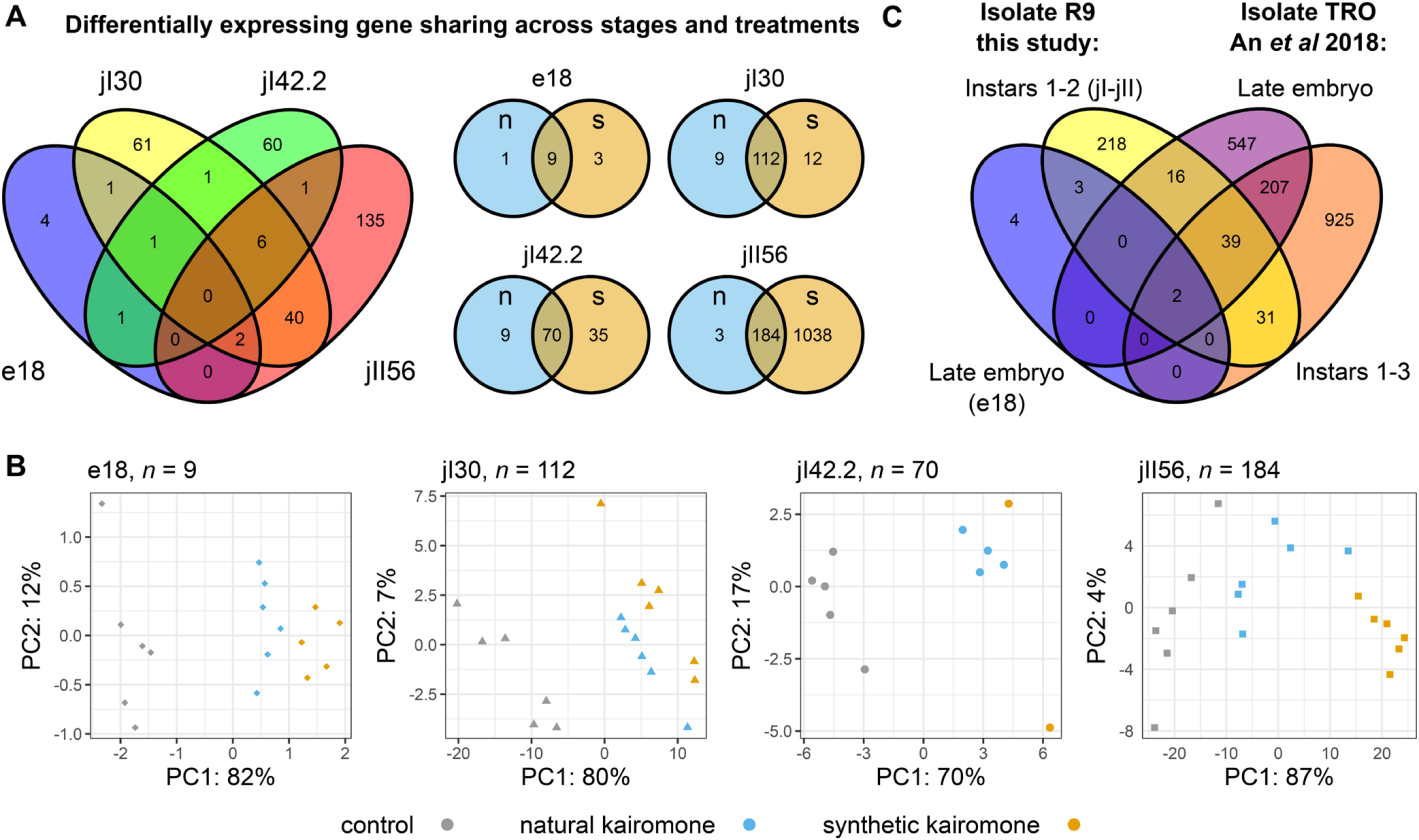
Summary of differentially expressed genes (DEGs) responding to the kairomone treatments. (A) Venn diagrams showing genes responding to both kairomones shared between stages (*left*) and genes with evidence of responding to both kairomone treatments or strong evidence of responding to one of the treatments in each stage (*right*). (B) Principal component analysis based on the expression levels of genes with evidence of differential expression in each stage; only the first two principal components are shown. Numbers indicate the corresponding numbers of genes per stage. (C) Similarities between the genes defined as responding to both kairomone treatments in this study to the list of the DEGs published by An et al. (2018). For the sake of comparison, only the ‘Late embryo’ and ‘1–3 Instars’ lists from An et al. (2018) were recruited and the jI30, jI42.2 and jII56 stages analyzed here were merged into a single ‘Instars 1–2’ list.

Inclusion of two kairomone treatments, the natural kairomone and the synthetic kairomone, in the experiment allowed us to assess how similar the responses to the two treatments are and to isolate the effect of the kairomone induction on gene expression from the influence of the other compounds produced by *Chaoborus*. Differential gene expression analysis was thus performed in a two‐stage manner to maximize the detection of genes responding to both treatments by first testing the general effect of kairomone treatment and then testing which treatments were responsible for the effect (see Section 2 and Figure S1B for details). This strategy yielded a total of 313 genes for which there was strong evidence of differential expression in at least one of the two kairomone treatments and at least suggestive evidence of differential gene expression in the other treatment across the four stages (Data S3, Figure 3A, Figure S4). At the same time, a considerable number of genes appeared differentially expressed only in one of the two kairomone treatments, with the synthetic kairomone consistently demonstrating higher numbers, especially in the jII56 stage (see Figure 3A). Nevertheless, log‐fold changes in the expression levels of the genes found to respond to both treatments were highly correlated between the two kairomone treatments in the same stage (Figure S5). The majority of the differentially expressed genes (DEGs) were up‐regulated and demonstrated a consistent direction of change across the different stages as indicated by high correlation coefficients in the log‐fold changes of the DEGs shared by the corresponding stages (see Figure S5). Ordination of the samples based on expression levels of DEGs demonstrated a clear separation of the samples into the three clouds corresponding to the three experimental conditions (Figure 3B), yet the differentiation of the synthetic‐kairomone‐treated jII56 clutches was most distinct, mirroring the clustering pattern based on the overall gene expression levels (see Figure 1B). A comparison of the lists of differentially expressed genes for the late embryonic stage and the combined juvenile stages to the results of An et al. (2018) revealed a group of 88 shared genes (Figure 3C), with two genes shared by all four gene lists (see below).

Noteworthy, the highest number of the differentially expressed genes was called in the latest stage, jII56, followed by the other post‐molt stage, jI30. We hypothesize that there is a genuine tendency for the post‐molt (jI30 and jII56) stages to have a stronger response to the kairomone treatments in comparison to the pre‐molt stages (e18 and jI42). It is likely that for the late embryonic stage (e18), despite the higher number of read data generated, a disproportionate number of DEGs went undetected in our analysis due to small fold changes and/or low expression levels, as at this early stage the kairomone response might be expected to be more subtle and restricted to particular organs or tissues. One compounding factor for the jI42 stage is the fact that only a subset of the samples was used for the differential gene expression analysis (see above), thus lowering the power of DEG detection. Nevertheless, when comparing the apparent log‐fold changes of DEGs between treatments and stages (see Figure S5), we noticed that the correlations between the fold‐changes of genes found to be differentially expressed in stages other than jI42 and their fold‐changes in jI42 were insignificant or weakly negative for both kairomone treatments. This indicates that most of the corresponding genes were indeed either non‐responsive or weakly responsive to the kairomone treatments in jI42.

**FIGURE 4:**
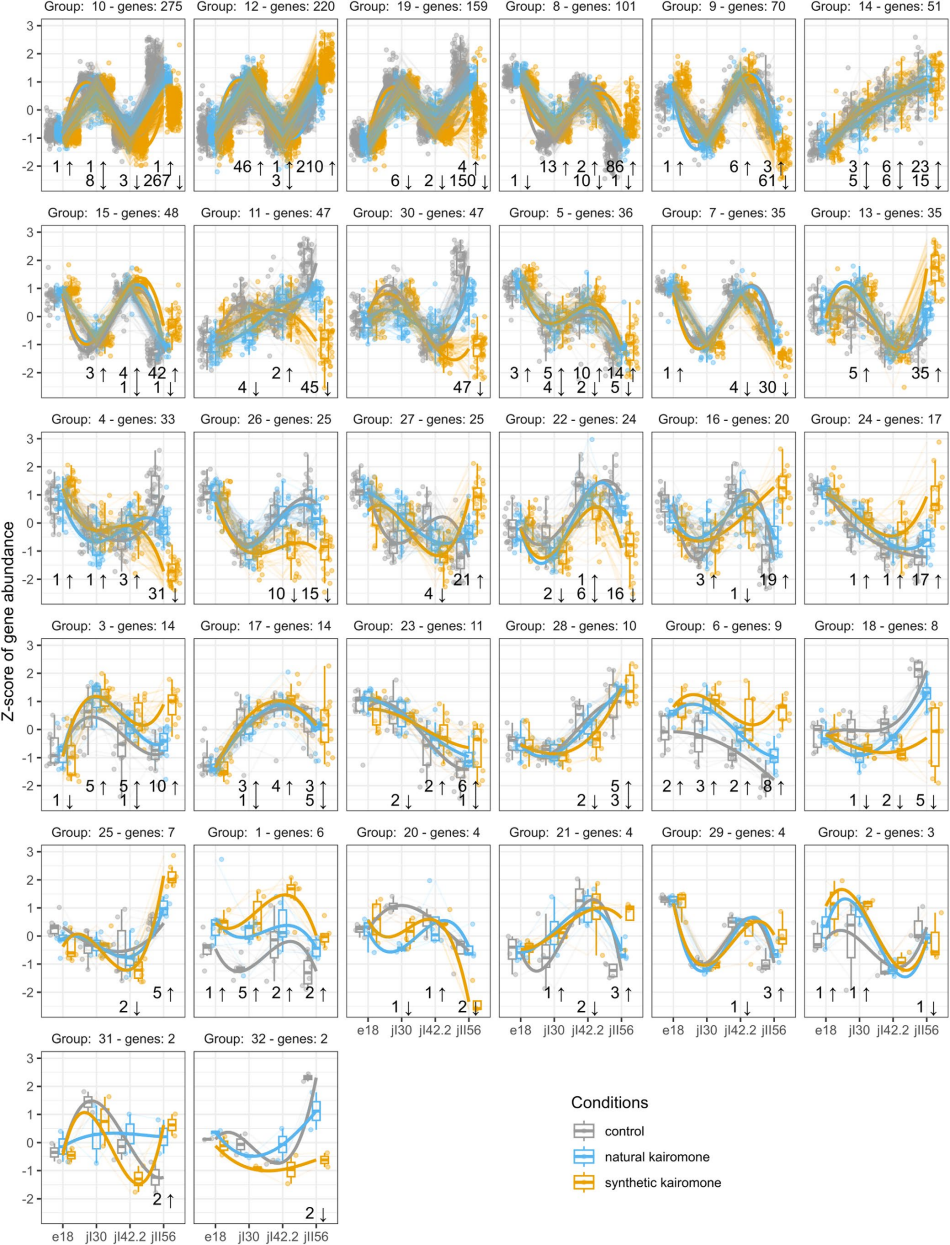
Expression patterns of the differentially expressed genes. rlog‐normalized counts were used to group genes according to their expression levels across developmental stages and treatments. Numbers below the plots refer to the number of genes differentially expressed in the corresponding stage and belonging to the group.

Several expression patterns of the DEGs across the developmental stage could be discerned with the largest groups of genes demonstrating one of the molting cycle‐dependent patterns in the treatment, as well as in the control clutches (Figure 4). At the same time, a minority of the DEGs showed clinal expression patterns across development (most prominently, groups 14, 23 and 28) or expression dynamics diverging between the control and the kairomone treatments (e.g., groups 11 and 27).

### 3.4 Differentially Expressed Genes Called in Multiple Stages

#### 3.4.1 Early‐Onset DEGs

**FIGURE 5:**
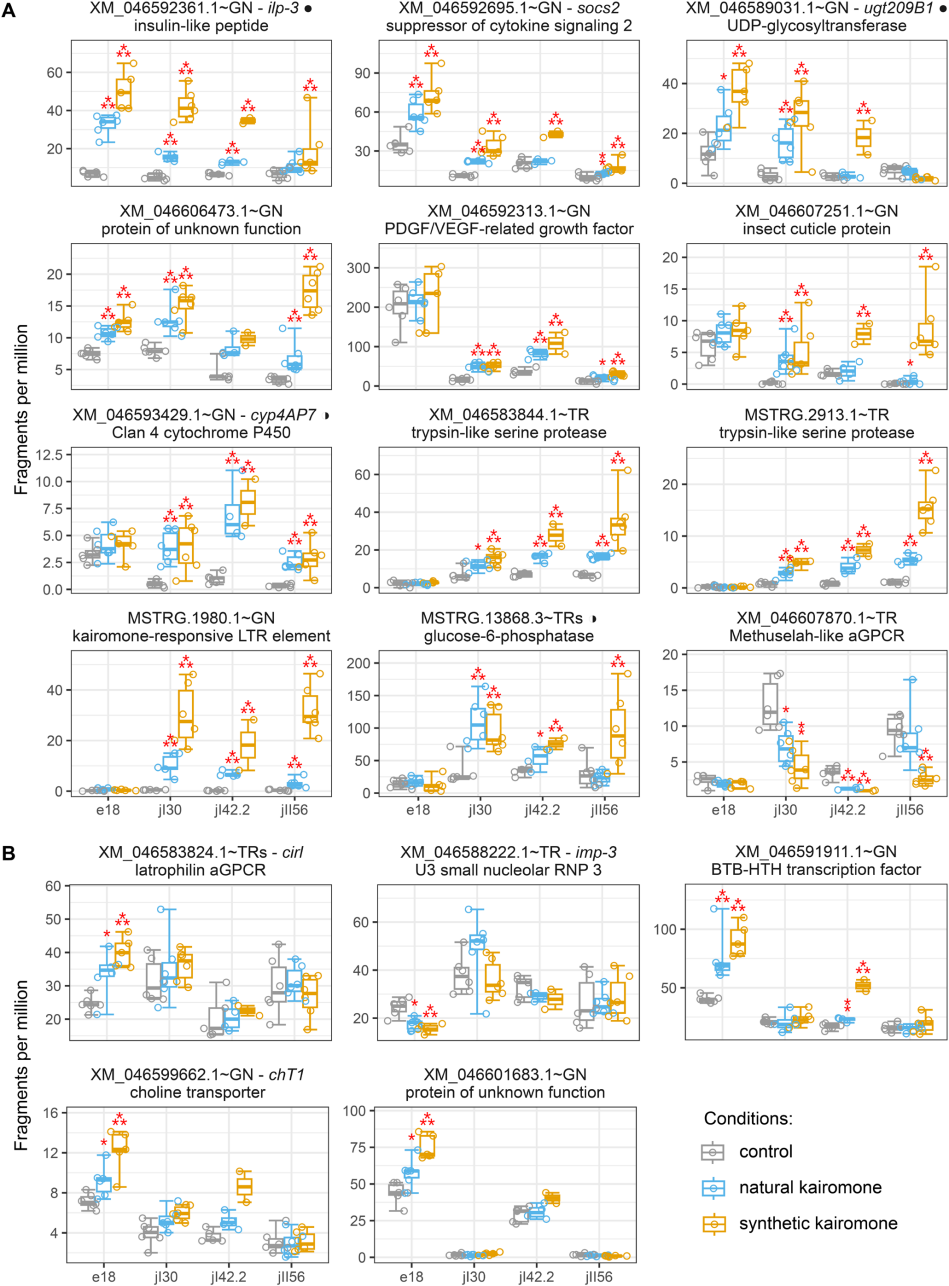
Expression levels of the top kairomone‐responding genes. (A) Transcripts found to be differentially expressed in at least three stages. (B) Other transcripts differentially expressed specifically in the embryonic stage (e18). Asterisks mark level of confidence: ⁂ and ⁑—likelihood ratio test (LRT) at the confirmation stage showing a significant contribution of the corresponding kairomone treatment to the full model with the overall FDR cutoff of 0.05 (⁂) or with raw *p*‐value cutoff of 0.01 (⁑) and the shrunk log_2_‐fold changes (LFC) exceed the threshold of 0.5 by absolute value; ⁎—same as ⁑ but |LFC| lower than 0.5. All of the marked cases pass the screening stage with FDR cutoff of 0.05 and have at least one of the two |LFC| values (for the natural and/or synthetic kairomone treatments, respectively) passing the 0.5 threshold. Genes correspond to groups of transcripts which can be told apart using the Tag‐Seq data (see Section 2). Filled circles and half‐circles next to the gene names indicate genes appearing on the list of the differentially expressed genes in An et al. (2018): Left half indicates genes from the ‘Late embryo’ list and right half—from the ‘1–3 Instars’ list.

Genes with differential expression at multiple stages are of particular interest as they might represent general regulators or indicators of stage‐independent physiological responses to the kairomone (Figure 5A). The most prominent of these were three genes with early onset of differential expression and gradual decline with age: (i) a gene for insulin‐like peptide (*ilp‐3*); (ii) an ortholog of suppressor of cytokine signaling 2 (*socs2*) whose expression pattern closely matches that of *ilp‐3* and (iii) *ugt209B1* coding for a UDP‐glycosyltransferase (UGT). *ilp‐3* and *ugt209B1* in particular are the two genes for which evidence of differential gene expression was found in both the late embryonic stage and the first juvenile stages here and in the study by An et al. (2018) on the TRO clone.

**FIGURE 6:**
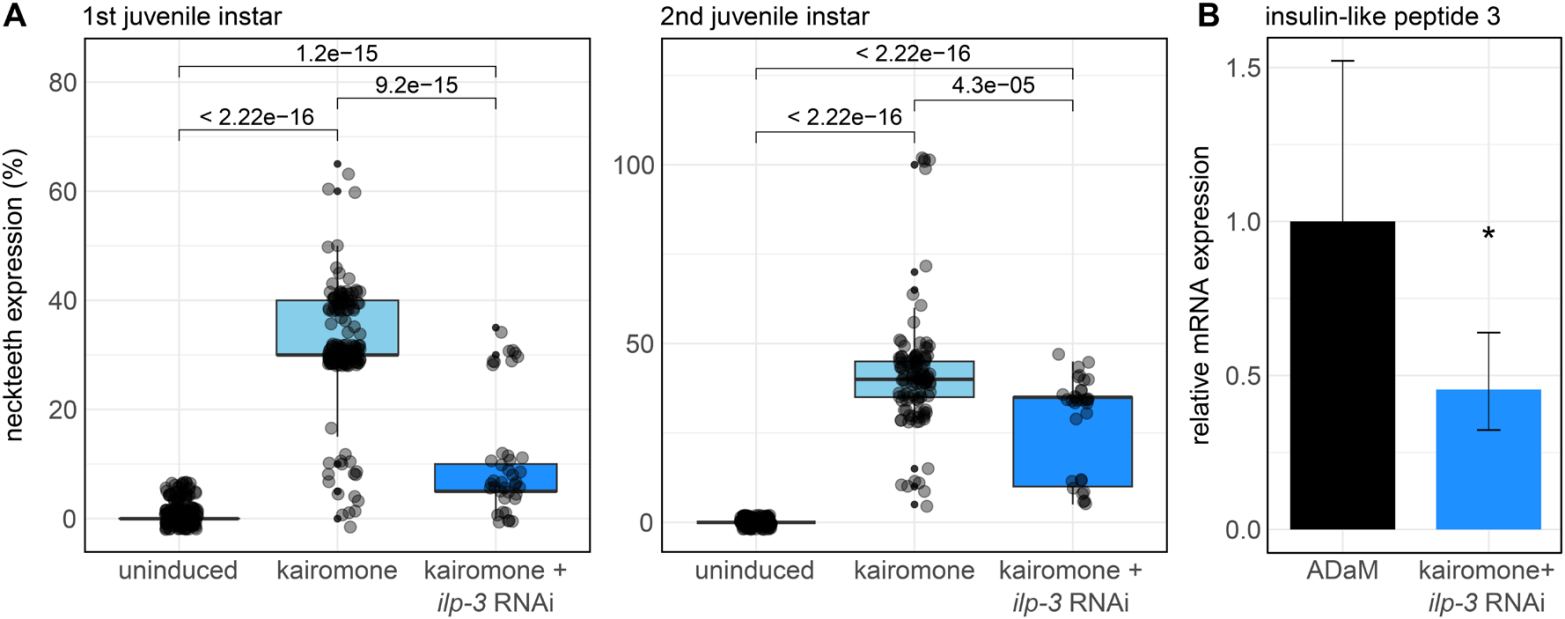
Knockdown of the kairomone‐responsive gene *ilp‐3* coding for an insulin‐like peptide, in *Daphnia pulex* R9. (A) Neckteeth phenotypes of the *ilp‐3* knockdown with the ILP_3_1 probe in the first (*left*) and second (*right*) juvenile instars. Numbers indicate *p*‐values of Bonferroni‐corrected multiple comparisons using Dunn's post hoc test. (B) qPCR results for the expression levels of *ilp‐3* in the control (ADaM) and the knockdown juveniles. Asterisks indicate *t*‐test *p*‐values < 0.05.

The first of these genes codes for the insulin‐like peptide 3 (ILP‐3 in Boucher et al. (2010), aIGF‐2 in Veenstra (2020)) from the family of arthropod insulin‐like growth factors (aIGFs) (Veenstra 2020), which has been implicated in the response to *Chaoborus* kairomone in our previous pilot RNA‐Seq study (Rozenberg et al. 2015). *ilp‐3* was significantly up‐regulated in both kairomone treatments during the first three stages: the expression differences were most prominent in the embryonic stage, but gradually decreased towards the final time point. To test whether ILP‐3 might be involved in the development of neckteeth, we knocked down *ilp‐3* using double‐stranded RNA interference (dsRNAi) (Figure 6). The gene knockdown led to a significant decrease in the neckteeth expression by 64.3% ± 36.0% (mean ± SD) in comparison to the kairomone‐exposed control juveniles in the 1st instar and a less prominent reduction of 32.3% ± 34.9% in the 2nd instar (Figure 6A) with similar results obtained with an alternative probe (Figure S6). At the same time, a complete knockdown could not be achieved as the *ilp‐3* expression levels could be lowered only by 54.6% ± 49.2% (Figure 6B), indicating that a further reduction in *ilp‐3* levels might lead to a more drastic phenotype.

Interestingly, *ilp‐3* represents just one member of a small family of related genes in *Daphnia*. Whereas *Ceriodaphnia dubia* has one gene from this group, three aIGF genes, *ilp‐1*, *ilp‐3* and *ilp‐4*, are present in all *Daphnia* species resulting from two gene duplication events (Figure S7).

The third gene with an early onset of differential regulation, gradual decline and even down‐regulation in juvenile 2 stage is *ugt209B1*. This gene and several similar paralogs are related to the insect single‐copy UGT50 family (Ahn et al. 2014), although the multitude of these genes in *Daphnia* makes them more similar to the other multi‐copy UGT families found in insects (Ahn et al. 2012).

XM_046606473.1~GN coding for a protein with a conservative domain otherwise frequently associated with a C1q domain in related proteins from *Daphnia* (data not shown) belongs to the same group of genes with an early onset of relatively constant differential expression, but its function is unknown.

#### 3.4.2 Late‐Onset DEGs

Several DEGs demonstrate retention of prenatal expression under kairomone treatment in juveniles in that their expression levels are relatively high in the late embryonic stage and subsequently drop in the juveniles but the genes remain up‐regulated under kairomone treatments. These genes include: (i) XM_046592313.1~GN coding for a PDGF/VEGF‐related growth factor, (ii) XM_046607251.1~GN for a cuticle protein (only weakly up‐regulated in the natural kairomone treatment in jI42.2 and iII56) and (iii) the cytochrome P450 gene *cyp4AP7* (see Figure 5A). Whereas the first two genes might be associated with tissue growth, the function of *cyp4AP7* is difficult to predict as it belongs to a *Daphnia*‐specific group of CYP genes without close relatives of known substrate specificity (Dermauw et al. 2020).

The second group of the genes with a later onset of differential expression have low expression levels in the embryonic stage and includes such heterogeneous cases as: (i) two transcripts mapping to genomic regions related to long terminal repeat (LTR) elements (MSTRG.1980.1 is shown in Figure 5A); (ii) two protease genes (see below); (iii) a gene for glucose‐6‐phosphatase and (iv) a systematically down‐regulated transcript XM_046607870.1~TR coding for a member of the Methuselah adhesion GPCR family similar to *Drosophila*'s *Mthl15* gene (see Figure 5A).

**FIGURE 7:**
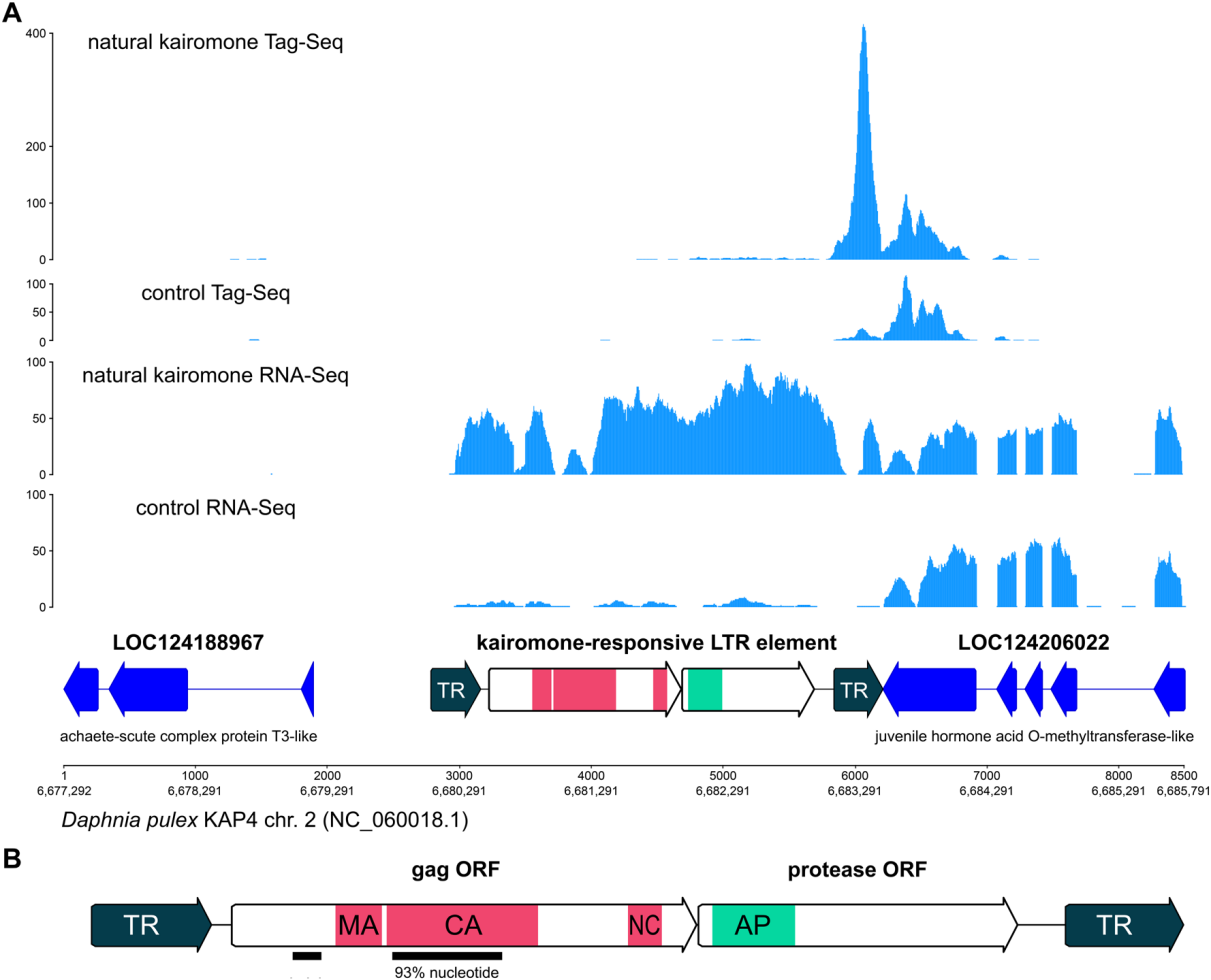
The *Chaoborus* kairomone‐responsive LTR element. (A) Genomic location of the LTR element (below) and the sequencing coverage around the locus in the Tag‐Seq (exemplified here by merged data from jI30 control and natural‐kairomone‐treated clutches) and RNA‐Seq data (merged data from two pilot natural kairomone experiments with pooled juveniles 1). (B) Structure of the LTR element with the terminal repeats (in orange) and the two ORFs. Homology regions are indicated for the ORFs: MA—matrix protein, CA—capsid protein, NC—putative nucleocapsid protein with Zinc‐finger domains; PR—retropepsin‐like aspartic protease. Indicated below are: An A‐rich region which putatively enabled detection of transcription from the locus using the Tag‐Seq protocol; two regions of lowered sequence identity between strains KAP4 and R9 responsible for the coverage gaps of the RNA‐Seq data.

Assembly and read mapping of RNA‐Seq demonstrated that the short transcript MSTRG.1980.1 is a fragment of a larger mRNA containing an entire LTR element with near‐zero expression in the control and high expression levels in the kairomone‐induced juveniles (Figure 7), and mapping of the Tag‐Seq data were consistent with a polyA tail present at its 3′ end. This kairomone‐responsive LTR element (KRLE) contains two open reading frames (ORFs) coding for: (i) a Ty3/Gypsy‐type gag polyprotein with intact matrix, capsid and nucleocapsid regions and (ii) a retropepsin. Phylogenetic analysis of gag sequences revealed a large clade of Ty3/Gypsy elements specific to Cladocera with varying gene architectures (Figure S8). Whereas most of these elements show signs of gene degradation, the majority of them still maintain ORFs for components of the pol polyprotein. It is thus noteworthy that the closest relatives of KRLE from KAP4 and R9 are similar LTR elements without *pol* genes found in other members of the *D. pulex* complex, although there are also closely related more complete retroelements retaining ORFs for the reverse transcriptase and integrase in *D. pulex* PA42 and *D. galeata* as well. The second transcript, MSTRG.10270.1, in KAP4 maps to a genomic region containing an isolated single copy of the terminal repeat closely related to that of KRLE.

### 3.5 Kairomone Induction in the Late Embryonic Stage

Genes which appear to be differentially expressed in both of the kairomone treatments exclusively or preferentially in the e18 clutches (Figure 5B) might be expected to have specific functions in the early response to the kairomone. Two such genes are constitutively expressed across the developmental stages, are up‐regulated in the kairomone‐treated embryos, and are functionally connected to neurohumoral regulation: *cirl* coding for a latrophilin‐family protein and *chT1* coding for a choline transporter.

Additionally, XM_046601683.1~GN coding for a protein from a *Daphnia*‐specific family with no recognizable homology to other protein families has an overall low expression level in j30 and jII56, but is slightly up‐regulated in the kairomone‐induced e18 embryos and its expression levels are also elevated in the kairomone‐treated jI42.2 clutches. A similar expression pattern was also observed for XM_046591911.1~GN which codes for a protein with a broad‐complex/tramtrack/bric‐à‐brac (BTB) domain and a divergent helix‐turn‐helix (HTH) DNA binding domain. Among *Dropsophila*'s BTB proteins, this domain composition is similar to that of the bab1 and bab2 developmental transcription regulators, although the HTH domain shows high similarity only to that of the BTB‐HTH protein CG3726 expressed in embryonic brain and larval nervous system (FlyBase 2024).

Finally, *imp‐3* coding for the small nucleolar protein IMP3 was found to be down‐regulated in the kairomone treatments, albeit with low fold differences. Interestingly, we find the *imp‐3* expression to be variable not only between the treatments but also across the developmental stages, with its expression levels doubling after hatching.

### 3.6 Gene Set Enrichment and Overrepresentation Analyses: Signatures of Cuticle Restructuring

**FIGURE 8:**
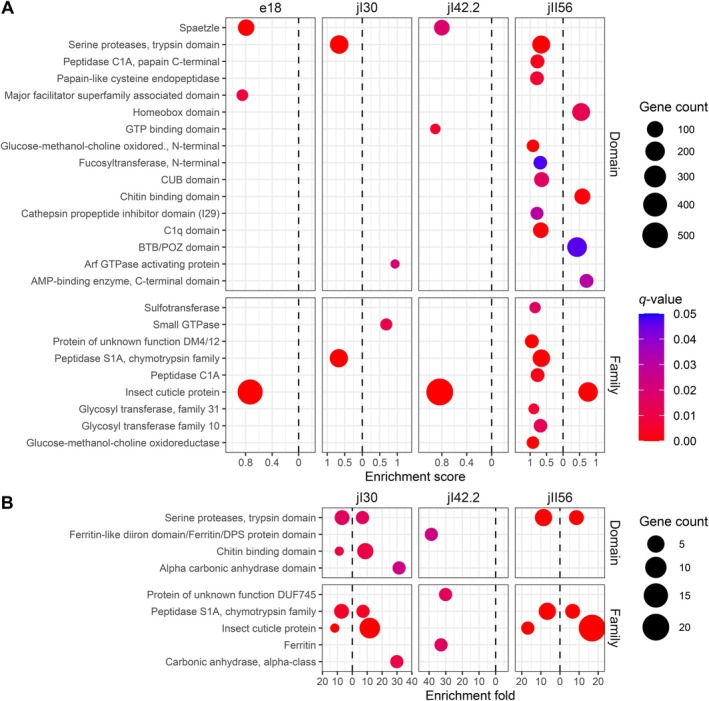
Enrichment analysis of protein domains and families among kairomone‐responsive transcripts. (A) Gene set enrichment analysis based on uncorrected log_2_‐fold changes in the synthetic kairomone treatment. (B) Over‐representation analysis for genes responding to both kairomone treatments. The size of the dots reflects the number of distinct transcripts in the corresponding category. The color of the dots corresponds to the *q*‐values. Dashed vertical lines separate down‐regulated transcripts (left) and up‐regulated transcripts (right).

For the analysis of higher‐level patterns in functions of DEGs we performed enrichment tests using predicted protein domains and family assignments of the *D. pulex* proteins. Proteins with chitin‐binding and protease(‐like) domains and the corresponding protein families, such as insect cuticle protein and chymotrypsin families are among the most consistently appearing groups of protein in both the gene set enrichment (GSE) and over‐representation (OR) analyses (Figure 8). The same functional groups appear to represent the most typical components of the cuticle proteome in *D. magna* (Otte et al. 2024), thus indicating that these proteins in our kairomone induction experiment are related to structural modifications of the cuticle. Further indication of the functional connection between protease(‐like) proteins and the cuticle is the appearance of fusion proteins with a protease‐like or protease inhibitor‐like and a chitin‐binding domain among differentially expressed genes, such as XM_046601842.1~GN (Figure S9) and others. As can be seen from the results of the enrichment analyses, the protein signatures that can be linked to the cuticle often demonstrate contrasting patterns of expression with some proteins having negative and some positive log‐fold changes in response to the kairomone treatment in the corresponding stages. Indeed, expression profiles of these proteins demonstrate a strong dependence on the molting cycle, with some proteins down‐ and some up‐regulated at the stages at which the corresponding transcripts peak (Figure S9). In addition, proteins with Spaetzle‐like domains found to be enriched in the GSE analysis in the e18 stage likewise also represent another one of the top five categories of the cuticle‐associated proteins in *D. magna* (Otte et al. 2024).

Although most of the genes in these categories appear to be differentially expressed at less than three stages and their expression levels follow the molting cycle (see Figure S9), the two related protease transcripts, MSTRG.2913.1 and XM_046583844.1, coding for proteins with trypsin‐like domains, represent an exception in this respect in showing a consistent increase in abundance in the kairomone treatments and thus might not be involved in the cuticle modification but in digestion (see Figure 5A and Figure S9).

## 4 Discussion

In the current work we analyzed transcriptomic responses in *D. pulex* to its invertebrate predator, larvae of the phantom midge *Chaoborus*, using two experimental treatments: the *Chaoborus* kairomone as part of the natural mix of compounds emitted by the predator and the dominant component of the kairomone mix synthesized chemically (Weiss et al. 2018). To our knowledge, this is the first study to analyze transcriptome responses of an aquatic organism to a kairomone synthesized in vitro. Our results demonstrate that the two kairomone treatments emit highly similar transcriptomic changes in *D. pulex* (see Figure 3B and Figure S5), thus confirming that fatty acid conjugates of L‐glutamine excreted by the predator are responsible for triggering the complex anti‐predatory responses in these crustaceans.

Gene expression analysis on the level of individual clutches across different stages, as opposed to large pools of individuals as used in previous experiments on anti‐predatory transcriptome responses in *Daphnia* (Miyakawa et al. 2010; Dennis et al. 2014; Otte et al. 2015; Rozenberg et al. 2015; An et al. 2018), provided us with the opportunity to study inter‐clutch variation in gene expression across the different developmental stages. In this respect, the experimental design used here is very different from the one utilized in the non‐replicated RNA‐Seq study of An et al. (2018) in which control and *Chaoborus*‐exposed pools of late embryos and juveniles spanning multiple stages were analyzed. The strength of our strategy lies in the fact that only individuals of exactly the same age and under the same maternal effects are pooled which allows the assessment of inter‐individual (inter‐clutch) variation. Indeed, this has allowed us to detect the finer structure in the jI42 stage (see Figures 1B and 2A) and detect an outlier e18 clutch. On the other hand, analysis of individual clutches forced us to compromise between sample sizes and sequencing depth which might be the reason behind the relatively low number of differentially expressed genes revealed in our study. To base our conclusions on a solid basis, we primarily focused on general trends in gene expression and on particular genes showing differential expression across multiple stages. It is also important to mention that for gene expression quantification, in contrast to the previous studies, we used a restriction‐independent Tag‐Seq protocol (Rozenberg et al. 2016). Whereas digital gene expression approaches, like Tag‐Seq, are in general statistically more powerful in differential expression analysis than RNA‐Seq (Hong et al. 2011), they yield little to no isoform resolution—even when relying on random shearing.

Our results demonstrate that gene expression patterns are highly dynamic in *Daphnia*'s development but that many of the genes involved in the transcriptomic responses to the kairomone production follow the molting cycle as well, leading to a bias in the number of the differentially expressed genes called in the different points along the molting cycle. Consequently, we find an abundance of genes coding for components of the cuticle among the kairomone‐responsive genes. Differential expression of such genes is stage‐dependent indicating that the cuticle undergoes a profound restructuring and not a linear increase in abundance of its components. Indeed, in *D. pulex* , besides the cuticular neckteeth, kairomone induction leads to such cuticular modifications as increased thickness and a higher number of layers in the procuticle, altogether leading to a higher cuticular stability (Kruppert et al. 2017). Similarly, the pillars connecting the two layers of integument of daphnia's carapace are also increased in numbers and distribution density in kairomone‐induced juveniles (Kruppert et al. 2016). Cuticle‐associated proteins are one of the prominent functional groups found among genes responding to invertebrate kairomones in daphniids with diverse anti‐predatory responses to predators such as *Ceriodaphnia cornuta* (Gu et al. 2022) and *D. magna* (Otte et al. 2014). Interestingly, studies on *D. ambigua* and *D. galeata* find cuticle‐associated proteins among differentially expressed genes under exposure to fish kairomones as well (Hales et al. 2017; Tams et al. 2020).

The short list of the genes with constitutive differential up‐regulation under kairomone treatment across the developmental stages and/or specifically in the embryonic stage is rich in neurohumoral factors. Most interestingly, we find that elevated expression and gradual decline of the gene coding for insulin‐like peptide ILP‐3 is the strongest correlate of the neckteeth production in the juvenile stages thus bearing witness to the involvement of insulin‐like signaling in the anti‐predatory responses. This aligns well with the role of some of the arthropod IGFs in phenotypic plasticity in insects, such as ornament development in the beetle *Gnatocerus cornutus* (Okada et al. 2019) and caste differentiation in the honey bee (Wolschin et al. 2010). Whereas our knockdown results (see Figure 6) suggest that ILP‐3 might be involved in the control of neckteeth production, conservation of this gene across the genus indicates that in *D. pulex* it is responsible for a systemic response to the kairomone and that it has other physiological roles as well. The role of SOCS2 vis‐a‐vis ILP‐3 signaling remains to be investigated. SOCS2 has been demonstrated to be a negative feedback regulator of growth hormone/IGF‐1 signaling in different tissues in vertebrates, facilitated by inhibition of STAT signaling and probably by binding to the IGF‐1 receptor (Dey et al. 1998; Greenhalgh et al. 2005; Isshiki et al. 2008; Ahmed and Farquharson 2010; Liu et al. 2018; Zhou et al. 2018). At the same time, the physiological functions of arthropod SOCS2 homologs range from innate immunity to modulation of ecdysteroid signaling and release of catecholamines (Zhang et al. 2010, 2013; Zhu et al. 2016; Yuan et al. 2020) and they have thus far not been associated with insulin signaling.

The PDGF/VEGF‐related factor differentially expressed in kairomone‐induced juveniles is a candidate for direct control of the tissue growth associated with the neckteeth: its generally high expression levels in the embryonic stage might be masking the more localized up‐regulation in the neck region. The BTB transcription regulator coded by XM_046591911.1~GN, which is differentially expressed under kairomone treatments in the two pre‐molt stages, is the prime candidate for a regulator of the morphological defense patterning given its ties to *Drosophila*'s developmental patterning regulators, although it does not demonstrate large fold changes in the kairomone‐treated embryos, indicating that it must have other functions as well. Among the other neurohumoral factors implicated in neckteeth production in the previous physiological and candidate‐gene studies (Miyakawa et al. 2010, 2015; Weiss, Kruppert, et al. 2012; Weiss et al. 2015), we find evidence for the involvement of neurotransmitter acetylcholine that enhances sensitivity to environmental cues, particularly predator‐induced defenses: *chT1*, which is part of the choline transporter family involved in choline uptake in the presynaptic terminal for acetylcholine biosynthesis, is up‐regulated in the kairomone‐induced embryos (and possibly in the jI42 stage). There is little indication of the differential expression of enzymes involved in production of other neurotransmitters, such as dopamine or glutamate (Miyakawa et al. 2015; Weiss et al. 2015; An et al. 2018), or expression of their receptors in the earliest stages of kairomone response in our data. As indicated above, this might be connected to the relatively subtle changes in expression levels of these genes. The up‐regulation of the latrophilin gene *cirl* in the kairomone‐induced embryos does nevertheless provide a link to dopamine as latrophilins in both the fruit fly and vertebrates have been connected to behavioral regulation and associated with dopamine signaling (van der Voet et al. 2016; Moreno‐Salinas et al. 2019). *dCirl* in *Drosophila* is expressed in the nervous system and plays a role in modulation of mechanosensation, and its silencing leads to hyperactivity (Scholz et al. 2015; van der Voet et al. 2016). It must be stressed that both *chT1* and *cirl* demonstrate very modest fold changes, which indicates that their functions are not restricted to the kairomone response.

Interestingly, alongside *ilp‐3* and *soc2*, we find *ugt209B1* coding for a UDP‐glycosyltransferase as one of the genes with the most consistent up‐regulation in the kairomone treatments (see Figure 5A). Its most plausible function is in kairomone detoxification, since xenobiotic detoxification is one of the typical functions of UGTs (Meech et al. 2019; Ahn and Marygold 2021). The finding of a gene coding for a putative detoxification enzyme among the kairomone‐responsive genes is not unexpected given kairomone's detectable toxicity at high concentrations according to our observations. Whereas it is tempting to connect *cyp4AP7* to detoxification as well, its high expression levels in the late embryo and lack of correlation with *ugt209B1* (see Figure 5A) speak against this hypothesis. It might be involved in the biosynthesis of endogenous infochemicals instead, for example, alarmones—by analogy to the involvement of some clan‐CYP4 enzymes in the biosynthesis of pheromones in moths (Rong et al. 2019).

Finding an LTR element among the kairomone‐responsive genes in the juvenile stages is rather unexpected. To the best of our knowledge, KRLE reported here is the first reported case of an LTR element differentially expressed under stress in an aquatic crustacean. Expression of some LTR elements is known to be triggered by stress conditions in *Drosophila*, where they are hypothesized to play regulatory roles in tissue regeneration (Milyaeva et al. 2023). The lack of the *pol* ORF is a characteristic KRLE shares, for example, with *arc* loci in dipterans and tetrapods which represent domesticated Ty3/Gypsy‐type *gag* genes that form virus‐like particles in neurons and regulate synaptic plasticity (Ashley et al. 2018; Pastuzyn et al. 2018; Avallone et al. 2023). The conservation of the terminal repeats, the varying gene architecture among closely‐related elements in different *Daphnia* (*Daphnia*) species and the presence of multiple copies in some of them (see Figure S8) indicate that, in contrast to *arc* loci, KRLE lost its *pol* ORF very recently and that it might be still mobile by hijacking retrotransposition machinery of related elements.

Two genes differentially expressed under the kairomone treatments in the juvenile stages might be connected to life‐history changes: the up‐regulated gene for glucose‐6‐phosphatase and the down‐regulated Methuselah‐like aGPCR. Various members of the Methuselah family in particular have been implicated in life history changes and stress resistance in insects (Araújo et al. 2013; Li et al. 2014), which leads us to the hypothesis that this gene might be one of the regulators of life‐history changes in *Daphnia* as well.

It is important to stress that the putative cellular determinants of neckteeth production are apparent in uninduced *Daphnia* (Beaton and Hebert 1997), thus many components of the pathway leading to expression of this morphological hallmark of the anti‐predatory defense, including the kairomone chemoreceptor, might be expressed constitutively and not change their expression levels upon exposure to the kairomone. Nevertheless, it is clear that our Tag‐Seq approach has its limitations: increase in sequencing depth and number of samples will increase the list of detected differentially expressed genes, although to solve the more intricate tissue‐specific changes in gene expression, single‐cell transcriptomic profiling would be necessary. In addition, most of the *Daphnia* genes are functionally annotated based on homology to other model arthropods, which together with the abundance of lineage‐specific gene families and protein domains makes it difficult to draw conclusions about gene functions and pathways. We hope that the accumulating gene expression datasets, including the one presented here, will lead to a better understanding of *Daphnia*'s gene functions and interactions. Our findings contribute to the growing literature on daphniid genetic responses to environmental stress (see Ravindran et al. 2019) and highlight the importance of incorporating a fine‐grained developmental perspective into such studies.

## Author Contributions

A.R., L.C.W. and R.T. planned the study; A.R. performed kairomone‐induction experiments for Tag‐Seq and did library preparation; N.K. and U.J. sequenced the libraries; T.S. and L.C.W. did gene knockdown experiments and their analysis; A.R. performed the bioinformatic analyses and drafted the manuscript. All authors contributed to the discussion of the data and have read and approved the manuscript.

## Funding

This study was supported by Deutsche Forschungsgemeinschaft (WE6019/2‐2).

## Conflicts of Interest

The authors declare no conflicts of interest.

## Supporting information


**Data S1:** List of the sequenced samples with metadata on indexing, sequencing batches, raw and deduplicated read counts and spike‐in mixes.

**Data S2:** DESeq2 quantification results (fragments per million values for each sample).

**Data S3:** Results of the differential gene expression analysis.

**Figure S1:** Details of the hypothesis testing procedure used for differential gene expression analysis. (A) Calculation of the molt phase covariate used to compensate for the asynchrony between clutches belonging to the same stage. (B) The two‐phase hypothesis testing and the subsequent binning of genes according to the evidence of differential expression (DE).
**Figure S2:** Proportion of PCR duplicates in the sequenced Tag‐Seq libraries as a function of the number of the reads.
**Figure S3:** Quantification of ExFold spike‐in transcripts in the Tag‐Seq samples. (A) ExFold transcript concentrations and their normalized Tag‐Seq read counts. (B) Correlations in the estimated ExFold transcript abundance between samples with the same mix (1 and 2, respectively). (C) Correlations between log‐fold differences in the abundance of ExFold transcripts predicted from pairs of samples assigned to mixes 1 and 2, respectively, and the expected log‐fold differences. Only transcripts with at least 5 reads were considered.
**Figure S4:** MA plots for the two kairomone treatments across the four developmental stages. Each dot represents a gene (transcript group, see Section 2) with colored dots corresponding to genes passing one of the significance criteria as detailed to the right.
**Figure S5:** Correlations between unshrunk log_2_‐fold changes (LFC) in expression levels of genes found to respond to the kairomone treatments. Each dot represents a transcript found to be differentially expressed in one (blue) or both (red) of the corresponding kairomone‐stage combinations. For both groups of transcripts numbers in the left upper corner represent correlation coefficients when significant. |LFC| values exceeding 6.0 are capped.
**Figure S6:** Knockdown of *ilp‐3* using the alternative RNAi probe ILP_3_2. Neckteeth phenotypes of the *ilp‐3* knockdown in the first (*left*) and second (*right*) juvenile instars. Numbers indicate *p*‐values of Bonferroni‐corrected multiple comparisons using Dunn's post hoc test.
**Figure S7:** Arthropod insulin‐like growth factors (aIGFs) in *Daphnia*. Left: Phylogenetic tree of the three aIGF genes, ILP‐1, ILP‐3 and ILP‐4 in *Daphnia* (*Daphnia*) (blue labels) and *Daphnia* (*Ctenodaphnia*) (gray labels). Black dots indicate branches with ultra‐fast bootstrap support values ≥ 90. The tree is rooted by sequences from non‐daphniid branchiopods (not shown). Monophyly of ILP‐3 was enforced by constrained reconstruction (see Section 2). Right: Predicted organization of the amino acid sequences of the three hormones and their genomic context with examples from the two *Daphnia* subgenera. SP—signal peptide, B and A—putative B‐ and A‐chains, C—C‐peptide, E—C‐terminal extension. The intercalating red bars indicate potential cleavage sites at conserved mono‐ and di‐basic residues. Predicted disulfide bridges between conserved cysteine residues are indicated as S–S (in yellow).
**Figure S8:** Phylogenetic analysis and architecture of the LTRs from the clade of retrotransposons related to the kairomone‐responsive LTR element in *Daphnia pulex*. Phylogeny is based on the representative protein sequences of the gag polyprotein. Representatives were chosen based on 90%‐identity clusters (see Section 2) and numbers in parentheses indicate numbers of cluster members. The color of the labels reflects subgenus: *Daphnia* (*Daphnia*) (blue labels), *Daphnia* (*Ctenodaphnia*) (gray labels) and other Cladocera (black). The structure of the corresponding loci is shown for each gag protein to the right: colored arrows indicate ORFs with the color showing the domain composition of the encoded proteins; black arrowheads indicate the presence of terminal repeats; red asterisks indicate elements with ORFs showing signs of degradation. The clade containing the kairomone‐responsive LTR element in *D. pulex* R9 is highlighted in red. Branches with dots have ultra‐fast bootstrap support values ≥ 90.
**Figure S9:** Expression profiles of genes coding for protease(‐like) proteins, proteins with chitin‐binding domain and insect cuticle proteins responding to both kairomone treatments in at least two stages. Genes were selected based on the presence of InterPro signatures IPR001254, IPR000618, IPR043504, IPR001254, IPR001314, IPR002557 and IPR036508 and on significance of differential gene expression in both kairomone treatments in at least two developmental stages. See Figure 5 in the main text for explanation of the annotations.
**Table S1:** RNAi probes used for *ilp‐3* knockdown and the corresponding qPCR primers used to confirm the knockdowns. Probe‐specific primer binding regions are underlined and the T7 promoter overhangs are indicated in italics.

## Data Availability

The raw data were deposited in the NCBI SRA database (accession SRR4244255, BioProject PRJNA343077). Quantification results and differential gene expression results for analysis excluding jI42.1 clutches are included as Data S2 and S3. Gene annotations, other quantification results and additional data are available via the Zenodo repository doi:10.5281/zenodo.19219717. The code used for the analyses is available at https://github.com/evoeco/kairomone‐tagseq.
