# Supplementary material for: Ontogenetic Sequence of Differential Gene Expression in Predator‐Induced Daphnia pulex
Source: Mol Ecol. 2026 Jul 1;35(13):e70446. doi: 10.1111/mec.70446 (PMC13322756; doi:10.1111/mec.70446)
Supplement: Supplementary file 4 — Figure S1: Details of the hypothesis testing procedure used for differential gene expression analysis. (A) Calculation of the molt phase covariate used to compensate for the asynchrony between clutches belonging to the same stage. (B) The two‐phase hypothesis testing and the subsequent binning of genes according to the evidence of differential expression (DE). Figure S2: Proportion of PCR duplicates in the sequenced Tag‐Seq libraries as a function of the number of the reads. Figure S3: Quantification of ExFold spike‐in transcripts in the Tag‐Seq samples. (A) ExFold transcript concentrations and their normalized Tag‐Seq read counts. (B) Correlations in the estimated ExFold transcript abundance between samples with the same mix (1 and 2, respectively). (C) Correlations between log‐fold differences in the abundance of ExFold transcripts predicted from pairs of samples assigned to mixes 1 and 2, respectively, and the expected log‐fold differences. Only transcripts with at least 5 reads were considered. Figure S4: MA plots for the two kairomone treatments across the four developmental stages. Each dot represents a gene (transcript group, see Section 2) with colored dots corresponding to genes passing one of the significance criteria as detailed to the right. Figure S5: Correlations between unshrunk log2‐fold changes (LFC) in expression levels of genes found to respond to the kairomone treatments. Each dot represents a transcript found to be differentially expressed in one (blue) or both (red) of the corresponding kairomone‐stage combinations. For both groups of transcripts numbers in the left upper corner represent correlation coefficients when significant. |LFC| values exceeding 6.0 are capped. Figure S6: Knockdown of ilp‐3 using the alternative RNAi probe ILP_3_2. Neckteeth phenotypes of the ilp‐3 knockdown in the first (left) and second (right) juvenile instars. Numbers indicate p‐values of Bonferroni‐corrected multiple comparisons using Dunn's post hoc test. Figure S [file MEC-35-e70446-s002.pdf]

Ontogenetic sequence of differential gene expression in predator-induced *Daphnia pulex*

Supplemental Figures and Tables

Andrey Rozenberg[1,2 *], Linda C. Weiss[1], Tatjana Schwarz[1], Nancy Kühne[3], Uwe John[3], Ralph Tollrian[1]

(1) Department of Animal Ecology, Evolution and Biodiversity, Ruhr University Bochum, Bochum, Germany

(2) The Technion - Israel Institute of Technology, Haifa, Israel

(3) Alfred Wegener Institute, Bremerhaven, Germany

* Corresponding author: alephreish@gmail.com, andrey.rozen@technion.ac.il

This file includes:

- **Figures S1-9**
- **Table S1**

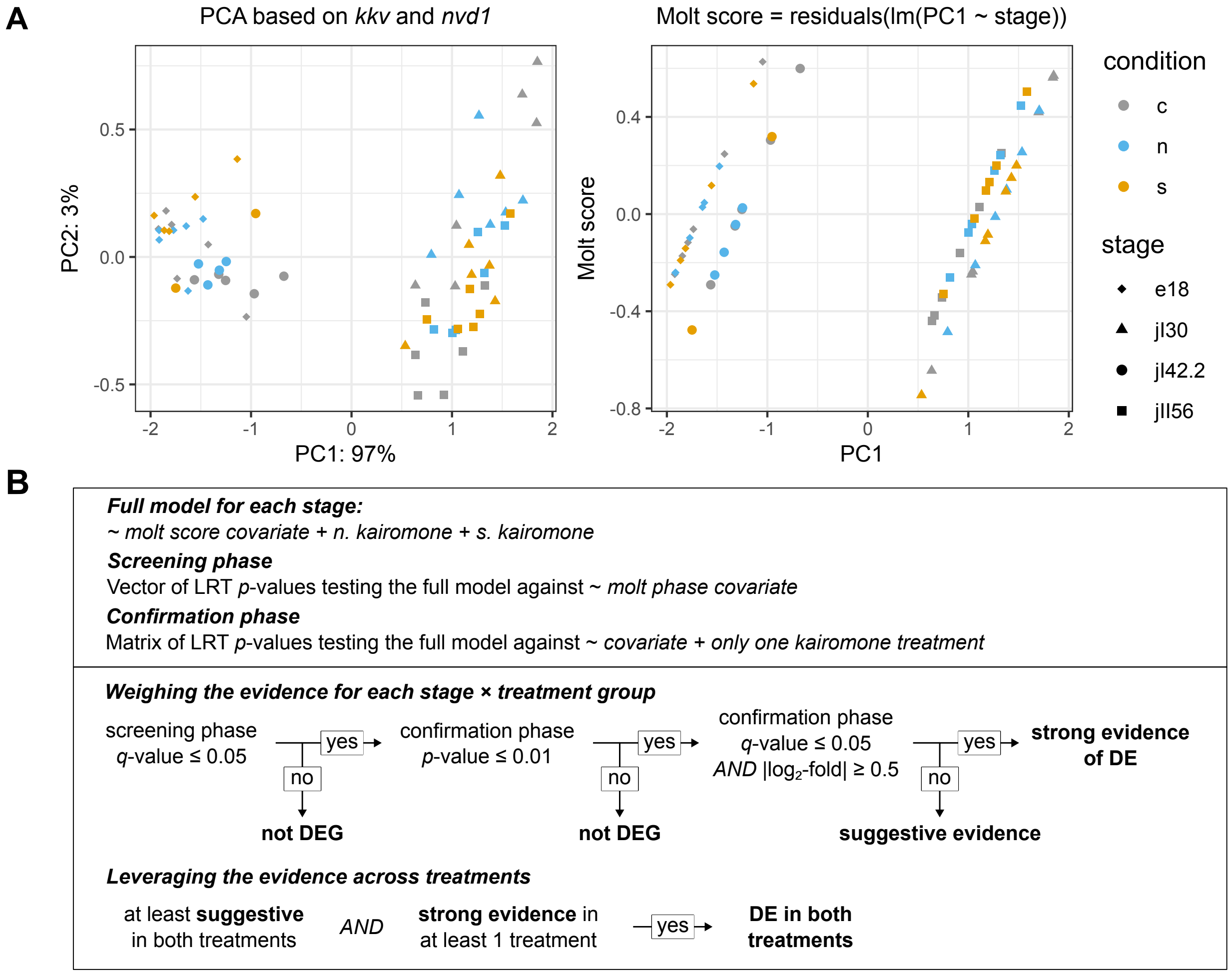

Figure S1. **Details of the hypothesis testing procedure used for differential gene expression analysis.** (A) Calculation of the molt phase covariate used to compensate for the asynchrony between clutches belonging to the same stage. (B) The two-phase hypothesis testing and the subsequent binning of genes according to the evidence of differential expression (DE).

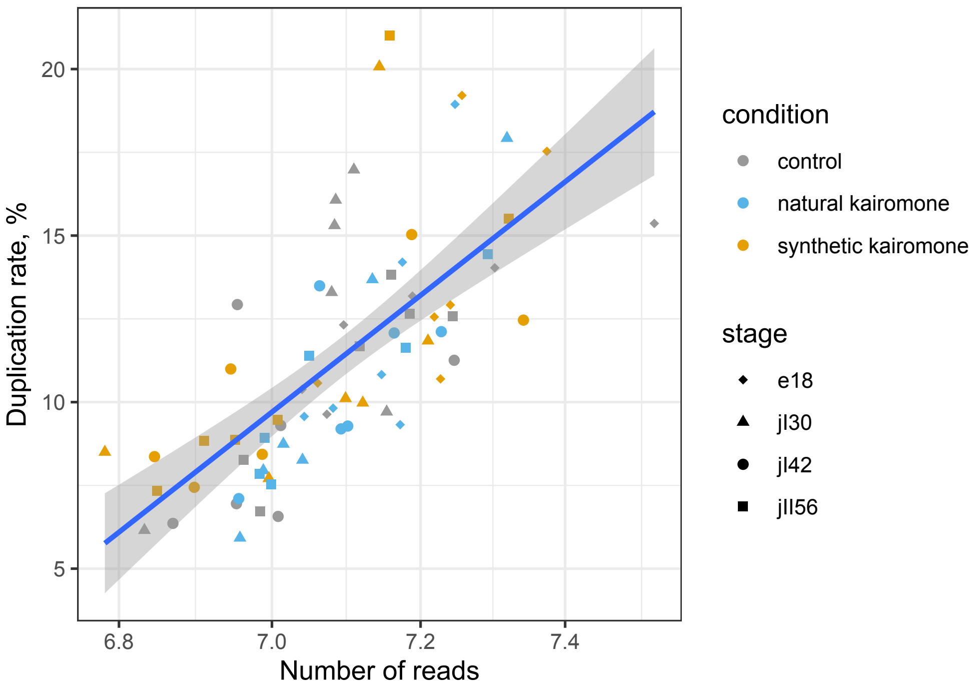

Figure S2. **Proportion of PCR duplicates in the sequenced Tag-Seq libraries as a function of the number of the reads.**

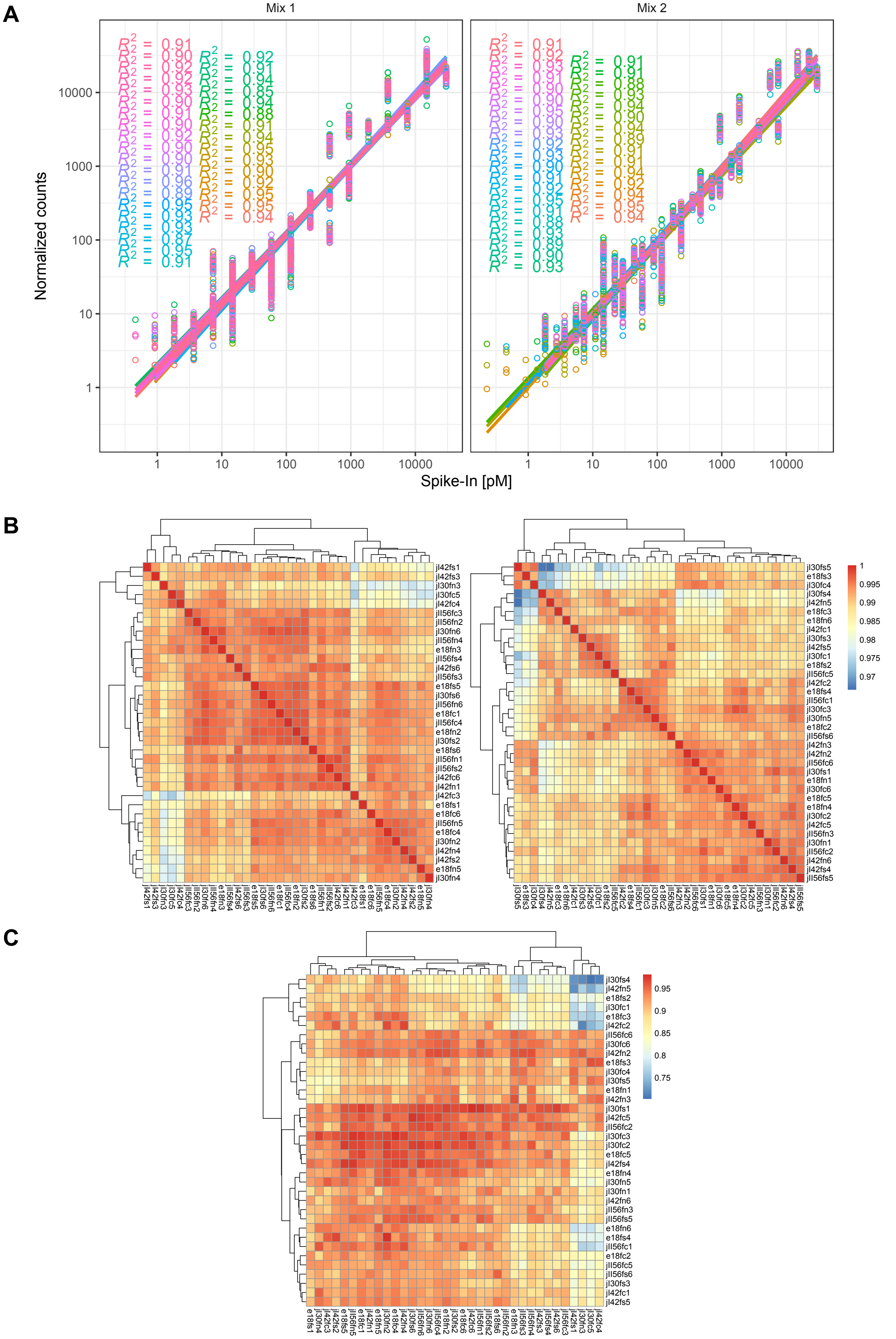
A
Mix 1
Mix 2
Normalized counts
Spike-In [pM]
B
C

Figure S3. **Quantification of ExFold spike-in transcripts in the Tag-Seq samples**. (A) ExFold transcript concentrations and their normalized Tag-Seq read counts. (B) Correlations in the estimated ExFold transcript abundance between samples with the same mix (1 and 2, respectively). (C) Correlations between log-fold differences in the abundance of ExFold transcripts predicted from pairs of samples assigned to mixes 1 and 2, respectively, and the expected log-fold differences. Only transcripts with at least 5 reads were considered.

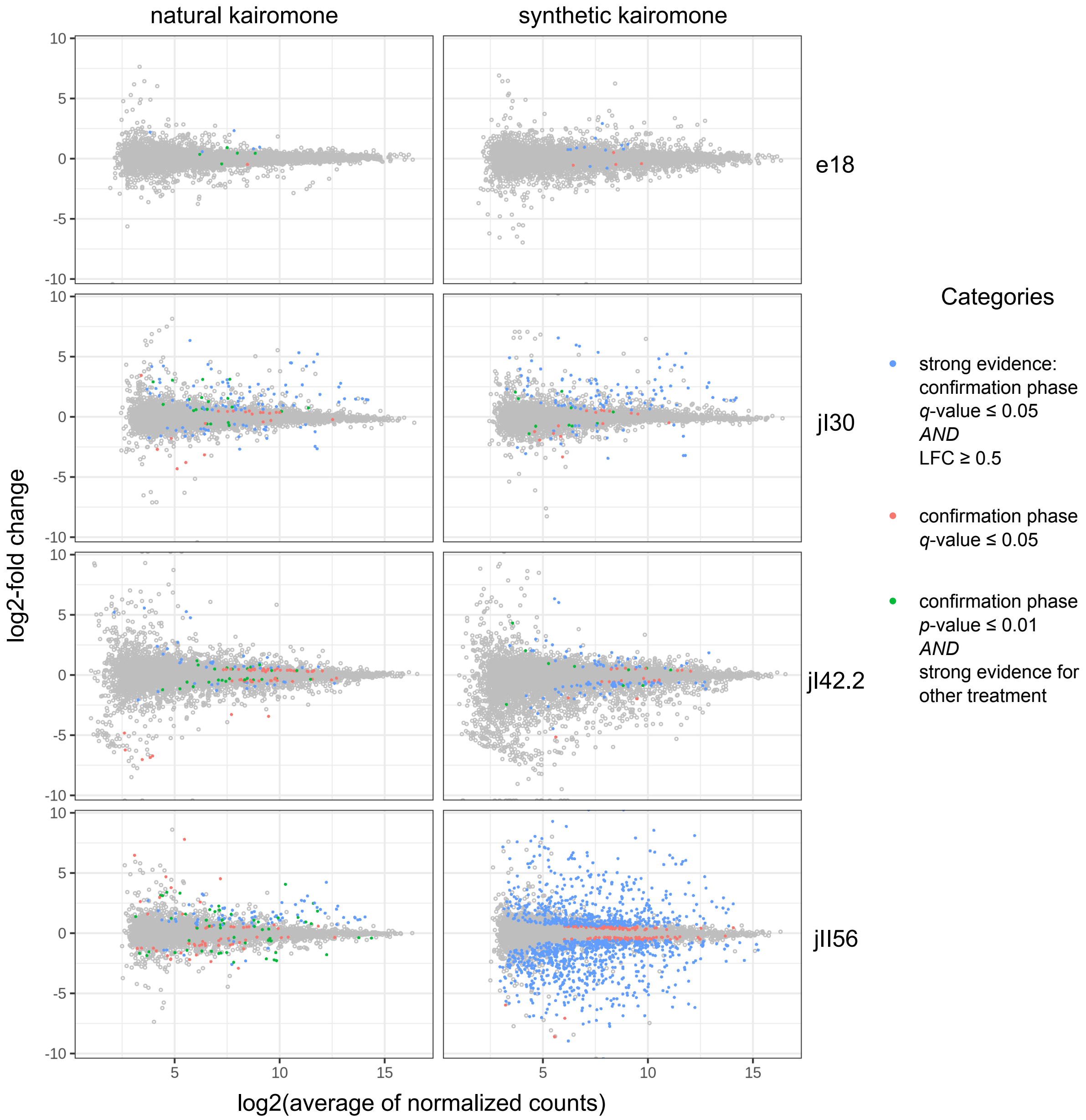

Figure S4. **MA plots for two kairomone treatments across the four developmental stages.** Each dot represents a gene (transcript group, see Materials and methods) with colored dots corresponding to genes passing one of the significance criteria as detailed to the right.

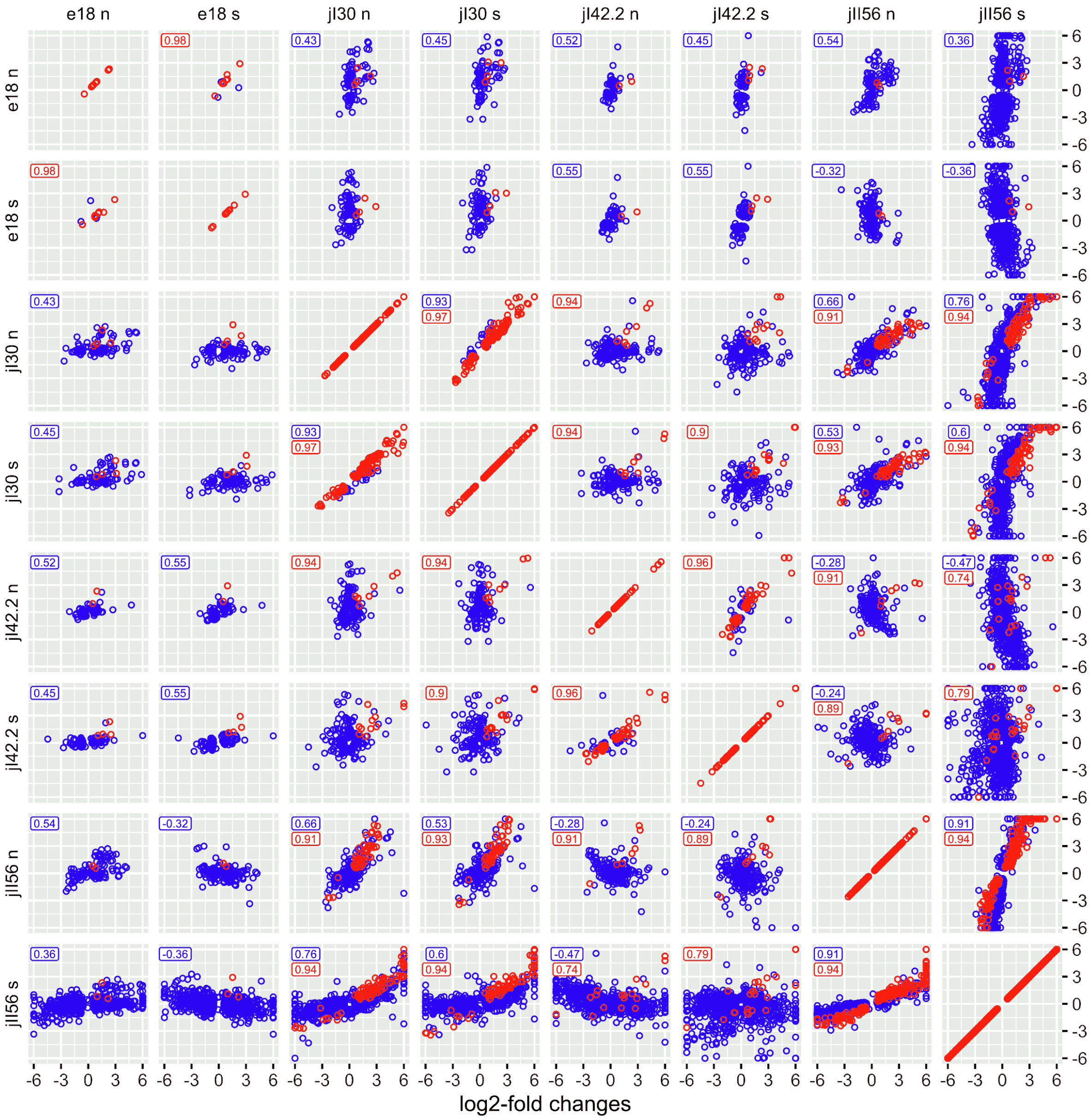

Figure S5. **Correlations between unshrunk log2-fold changes (LFC) in expression levels of genes found to respond to the kairomone treatments.** Each dot represents a transcript found to be differentially expressed in one (blue) or both (red) of the corresponding kairomone-stage combinations. For both groups of transcripts numbers in the left upper corner represent correlation coefficients when significant. |LFC| values exceeding 6.0 are capped.

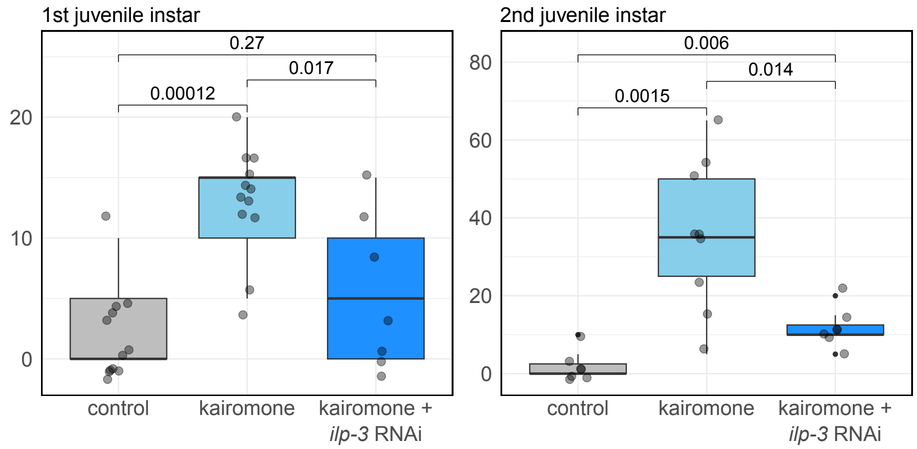

Figure S6**. Knockdown of *ilp-3* using the alternative RNAi probe ILP_3_2.** Neckteeth phenotypes of the *ilp-3* in the first (*left*) and second (*right*) juvenile instars. Numbers indicate *p*-values of Bonferroni-corrected multiple comparisons using Dunn's post hoc test.

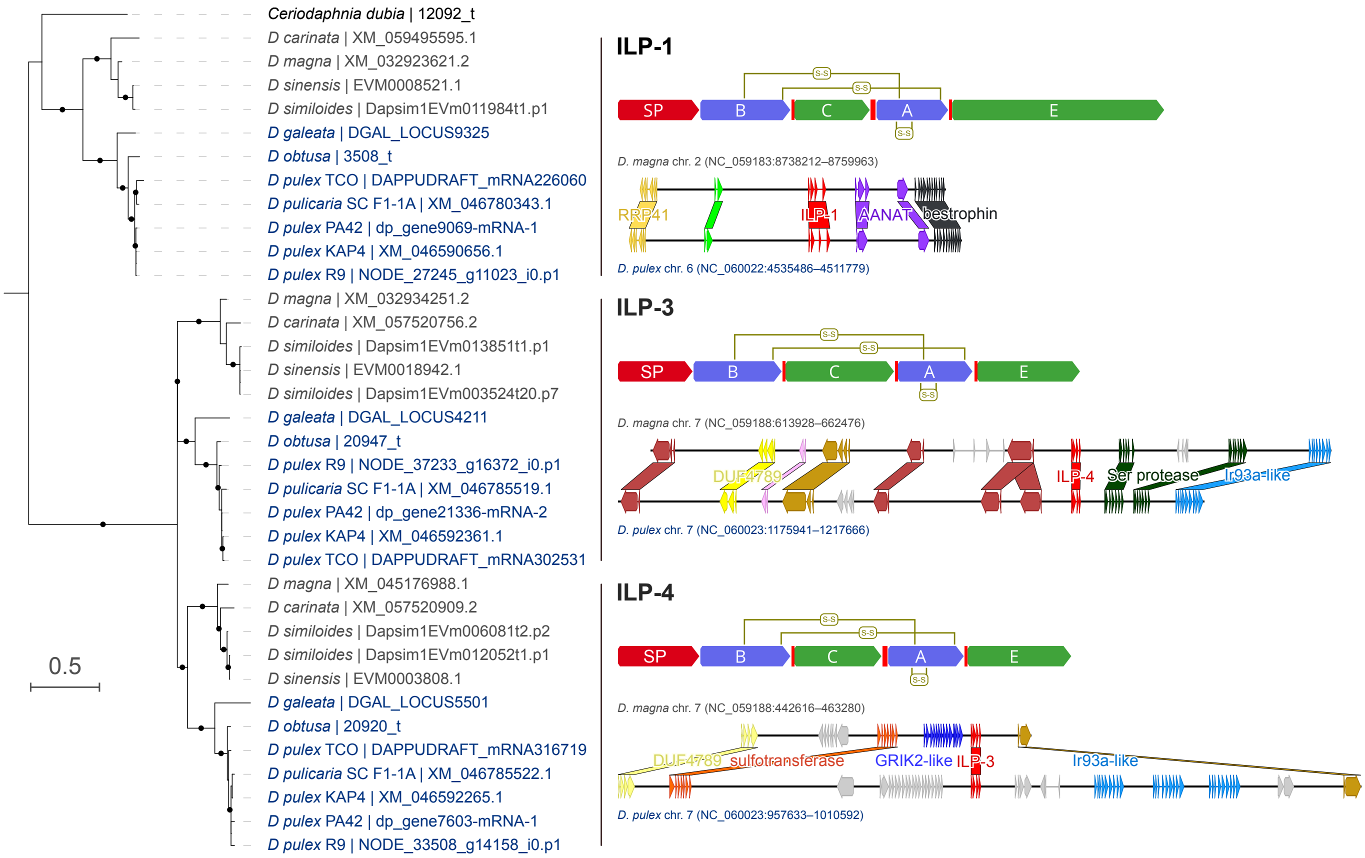

Figure S7. **Arthropod insulin-like growth factors (aIGFs) in *Daphnia*.** Left: Phylogenetic tree of the three aIGF genes, ILP-1, ILP-3 and ILP-4 in *Daphnia* (*Daphnia*) (blue labels) and *Daphnia* (*Ctenodaphnia*) (gray labels). Right: Predicted organization of the amino acid sequences of the three hormones and their genomic context with examples from the two *Daphnia* subgenera. SP — signal peptide, B and A — putative B- and A-chains, C — C-peptide, E — C-terminal extension. The intercalating red bars indicate potential cleavage sites at conserved mono- and di-basic residues. Predicted disulfide bridged between conserved cysteine residues are indicated as S-S (in yellow).

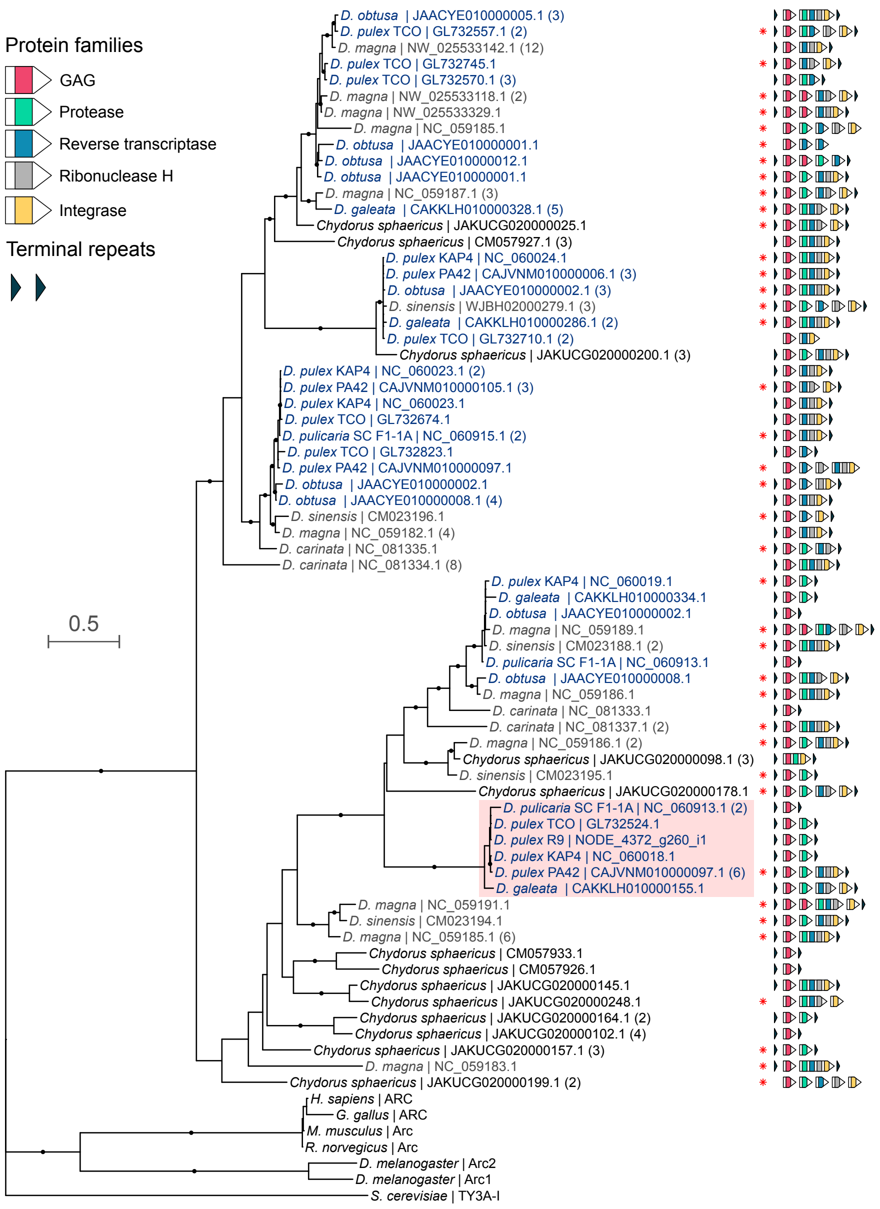
Protein families
GAG
Protease
Reverse transcriptase
Ribonuclease H
Integrase
Terminal repeats
0.5
D. obtusa | JAACYE010000005.1 (3)
D. pulex TCO | GL732557.1 (2)
D. magna | NW_025533142.1 (12)
D. pulex TCO | GL732745.1
D. pulex TCO | GL732570.1 (3)
D. magna | NW_025533118.1 (2)
D. magna | NW_025533329.1
D. magna | NC_059185.1
D. obtusa | JAACYE010000001.1
D. obtusa | JAACYE010000012.1
D. obtusa | JAACYE010000001.1
D. magna | NC_059187.1 (3)
D. galeata | CAKKLH010000328.1 (5)
Chydorus sphaericus | JAKUCG020000025.1
Chydorus sphaericus | CM057927.1 (3)
D. pulex KAP4 | NC_060024.1
D. pulex PA42 | CAJVNM010000006.1 (3)
D. obtusa | JAACYE010000002.1 (3)
D. sinensis | WJBH02000279.1 (3)
D. galeata | CAKKLH010000286.1 (2)
D. pulex TCO | GL732710.1 (2)
Chydorus sphaericus | JAKUCG020000200.1 (3)
D. pulex KAP4 | NC_060023.1 (2)
D. pulex PA42 | CAJVNM010000105.1 (3)
D. pulex KAP4 | NC_060023.1
D. pulex TCO | GL732674.1
D. pulicaria SC F1-1A | NC_060915.1 (2)
D. pulex TCO | GL732823.1
D. pulex PA42 | CAJVNM010000097.1
D. obtusa | JAACYE010000002.1
D. obtusa | JAACYE010000008.1 (4)
D. sinensis | CM023196.1
D. magna | NC_059182.1 (4)
D. carinata | NC_081335.1
D. carinata | NC_081334.1 (8)
D. pulex KAP4 | NC_060019.1
D. galeata | CAKKLH010000334.1
D. obtusa | JAACYE010000002.1
D. magna | NC_059189.1
D. sinensis | CM023188.1 (2)
D. pulicaria SC F1-1A | NC_060913.1
D. obtusa | JAACYE010000008.1
D. magna | NC_059186.1
D. carinata | NC_081333.1
D. carinata | NC_081337.1 (2)
D. magna | NC_059186.1 (2)
Chydorus sphaericus | JAKUCG020000098.1 (3)
D. sinensis | CM023195.1
Chydorus sphaericus | JAKUCG020000178.1
D. pulicaria SC F1-1A | NC_060913.1 (2)
D. pulex TCO | GL732524.1
D. pulex R9 | NODE_4372_g260_i1
D. pulex KAP4 | NC_060018.1
D. pulex PA42 | CAJVNM010000097.1 (6)
D. galeata | CAKKLH010000155.1
D. magna | NC_059191.1
D. sinensis | CM023194.1
D. magna | NC_059185.1 (6)
Chydorus sphaericus | CM057933.1
Chydorus sphaericus | CM057926.1
Chydorus sphaericus | JAKUCG020000145.1
Chydorus sphaericus | JAKUCG020000248.1
Chydorus sphaericus | JAKUCG020000164.1 (2)
Chydorus sphaericus | JAKUCG020000102.1 (4)
Chydorus sphaericus | JAKUCG020000157.1 (3)
D. magna | NC_059183.1
Chydorus sphaericus | JAKUCG020000199.1 (2)
H. sapiens | ARC
G. gallus | ARC
M. musculus | Arc
R. norvegicus | Arc
D. melanogaster | Arc2
D. melanogaster | Arc1
S. cerevisiae | TY3A-I

Figure S8. **Phylogenetic analysis and architecture of the LTRs from a clade of retrotransposons related to the kairomone-responsive LTR element in *D. pulex*.** Phylogeny is based on the representative protein sequences of the gag polyprotein. Representatives were chosen based on 90%-identity clusters (see Methods) and numbers in parentheses indicate numbers of cluster members. The color of the labels reflects subgenus: *Daphnia* (*Daphnia*) (blue labels), *Daphnia* (*Ctenodaphnia*) (gray labels) and other Cladocera (black). The structure of the corresponding loci is shown for each gag protein to the right: colored arrows indicate ORFs with the color showing the domain composition of the encoded proteins; black arrowheads indicate the presence of terminal repeats; red asterisks indicate elements with ORFs showing signs of degradation. The clade containing the kairomone-responsive LTR element in *D. pulex* R9 is highlighted in red. Branches with dots have ultra-fast bootstrap support values ≥90.

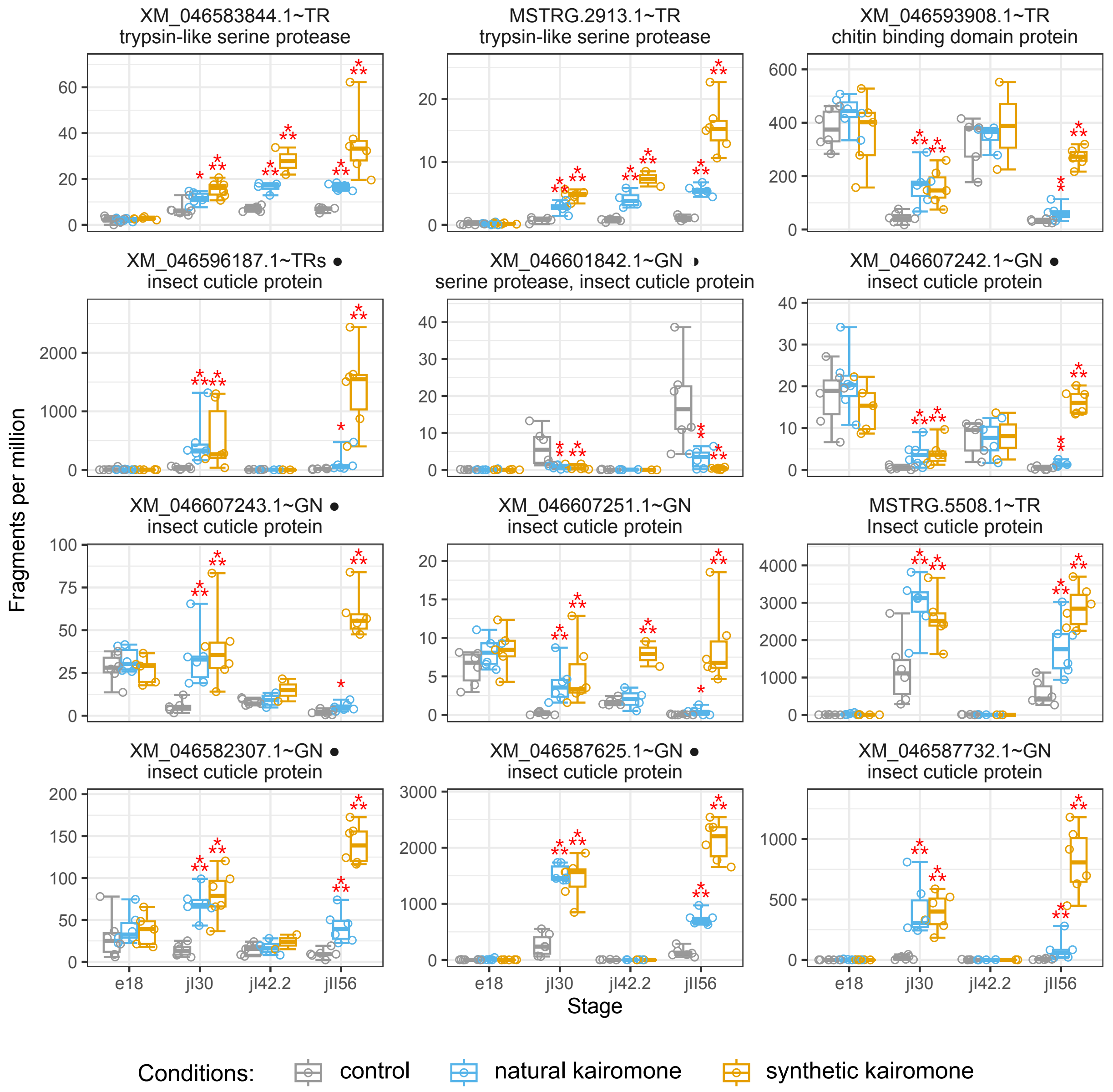

Figure S9. **Expression profiles of genes coding for protease(-like) proteins, proteins with chitin-binding domain and insect cuticle proteins responding to both kairomone treatments in at least two stages.** Genes were selected based on the presence of InterPro signatures IPR001254, IPR000618, IPR043504, IPR001254, IPR001314, IPR002557 and IPR036508 and on significance of differential gene expression in both kairomone treatments in at least two developmental stages. See Figure 5 in the main text for explanation of the annotations.

Table S1. **RNAi probes used for *ilp-3* knockdown and the corresponding qPCR primers used to confirm the knockdowns.** Probe-specific primer binding regions are underlined and the T7 promoter overhangs are indicated in italics.

Probe	Probe sequences	Probe length	qPCR primers
ILP_3_1	*TAATACGACTCACTATAGG*-TGTATTTTTTTACTGGACTGGCCACTTTCACGCTTGCCCGTCCACCTCAGGAAAATCAGCCAATGACGATCCGCTTCTGCGGACGTGATTTGATCAGAGCCATAGACGAGGTCTGCGTCGCAATCAAAAGCCCAGCAGCTTTTATCCATCCGGTGGCCCTCAATCAGTCGTCGTCCTTGGCTGACTCTGAATCAGCCGACACTCAGTTGA-*CCTATAGTGAGTCGTATTA*	211 bp	Forward: 5’-GCGTCAGTGTTGCGTGATTG-3’<br>Reverse: 5’-TCTCCATGTCGCGGTAGAAATTC-3
ILP_3_2	*TAATACGACTCACTATAGG*-GTGGACGGAATTGGAAGACAAGAAAAGCGATTCGCTTTTGCGTCAGTGTTGCGTGATTGGCTGCACGGAGAACGACTTGACTACTTTCTGCCAGATCGACAGGAATCAAATAATGGGAGCGGTGATGCATCTGAGGAGTCAAGAATGGGATATGGATTGGCTCTACGAATTTCTACCGCGACATGG-*CCTATAGTGAGTCGTATTA*	186 bp	
